# Insight into the Photodynamics of Photostabilizer Molecules

**DOI:** 10.1021/acs.jpca.2c05580

**Published:** 2022-11-02

**Authors:** Temitope T. Abiola, Benjamin Rioux, Sharanjit Johal, Matthieu M. Mention, Fanny Brunissen, Jack M. Woolley, Florent Allais, Vasilios G. Stavros

**Affiliations:** † Department of Chemistry, University of Warwick, Gibbet Hill Road, Coventry, CV4 7ALUnited Kingdom; § URD Agro-Biotechnologies Industrielles (ABI), CEBB, AgroParisTech, 51110, Pomacle, France

## Abstract

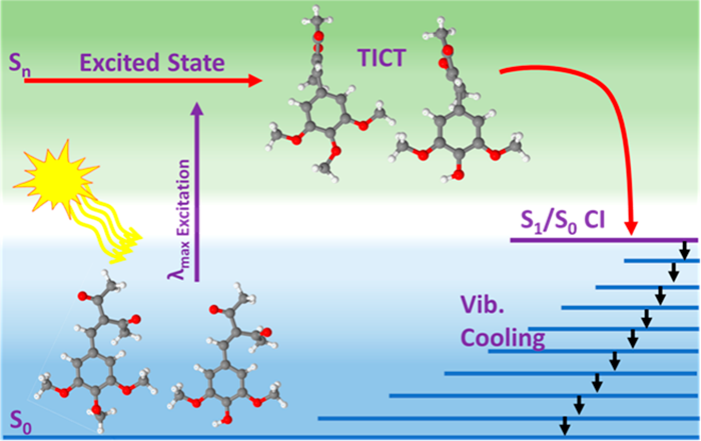

Solar exposure of avobenzone, one of the most widely used commercial UVA filters on the market, is known to cause significant degradation. This finding has fueled research into developing photostabilizer molecules. In an effort to provide insight into their stand-alone photoprotection properties, the excited state dynamics of the photostabilizer, 3-(3,4,5-trimethoxybenzylidene) pentane-2,4-dione (TMBP), and its phenolic derivative, 3-(4-hydroxy-3,5-dimethoxybenzylidene) pentane-2,4-dione (DMBP), were studied with ultrafast transient absorption spectroscopy. Solutions of TMPB and DMBP in ethanol and in an industry-standard emollient, as well as TMBP and DMBP deposited on synthetic skin mimic, were investigated. These experiments were allied with computational methods to aid interpretation of the experimental data. Upon photoexcitation, these photostabilizers repopulate the electronic ground state via nonradiative decay within a few picoseconds involving a twisted intramolecular charge transfer configuration in the excited state, followed by internal conversion and subsequent vibrational cooling in the ground state. This finding implies that, aside from acting as a photostabilizer to certain UV filters, TMBP and DMBP may offer additional photoprotection in a sunscreen formulation as a stand-alone UV filter. Finally, TMBP and DMBP could also find applications as molecular photon-to-heat converters.

## Introduction

The harmful effects of the overexposure to ultraviolet (UV) radiation from the Sun have been well-documented.^1−5^ UV radiation is commonly divided into three wavelength regions, namely, UVA (400–320 nm), UVB (320–280 nm), and UVC (280–100 nm).^6,7^ The UVC is filtered by the ozone layer, leaving UVB (∼5%) and UVA (∼95%) to account for the UV radiation at the Earth’s surface.^8,9^ Although UVA is less energetic compared to UVB, it is able to penetrate deeper into the human skin, reaching far into the dermis.^10,11^ The adverse effect of UVA radiation on human skin includes suppression of acquired immunity and production of harmful reactive oxygen species (ROS) that can cause DNA damage and subsequent skin cancer.^10,12^ To prevent skin damage and ultimately skin cancer, UVA filters are included in sunscreen formulations. Upon the application of sunscreen to the human skin, the UVA filters prevent the penetration of UVA radiation into the dermal layer of the skin which could result into the formation of ROS.^13^ Despite human skin requiring photoprotection against UVA, there is a sparsity of approved UVA filters. Importantly, the most widely used UVA filter avobenzone, lacks the long-term (up to 2 h after application) photostability required of a UV filter.^14,15^ The photoinstability of avobenzone is mediated by enol-keto tautomerization reaction following photoexcitation of the original enol form by solar radiation. The diketo photoproduct absorbs UVA radiation with less efficiency, resulting in reduced sunscreen efficacy upon exposure to UV radiation.

**Figure 1:**
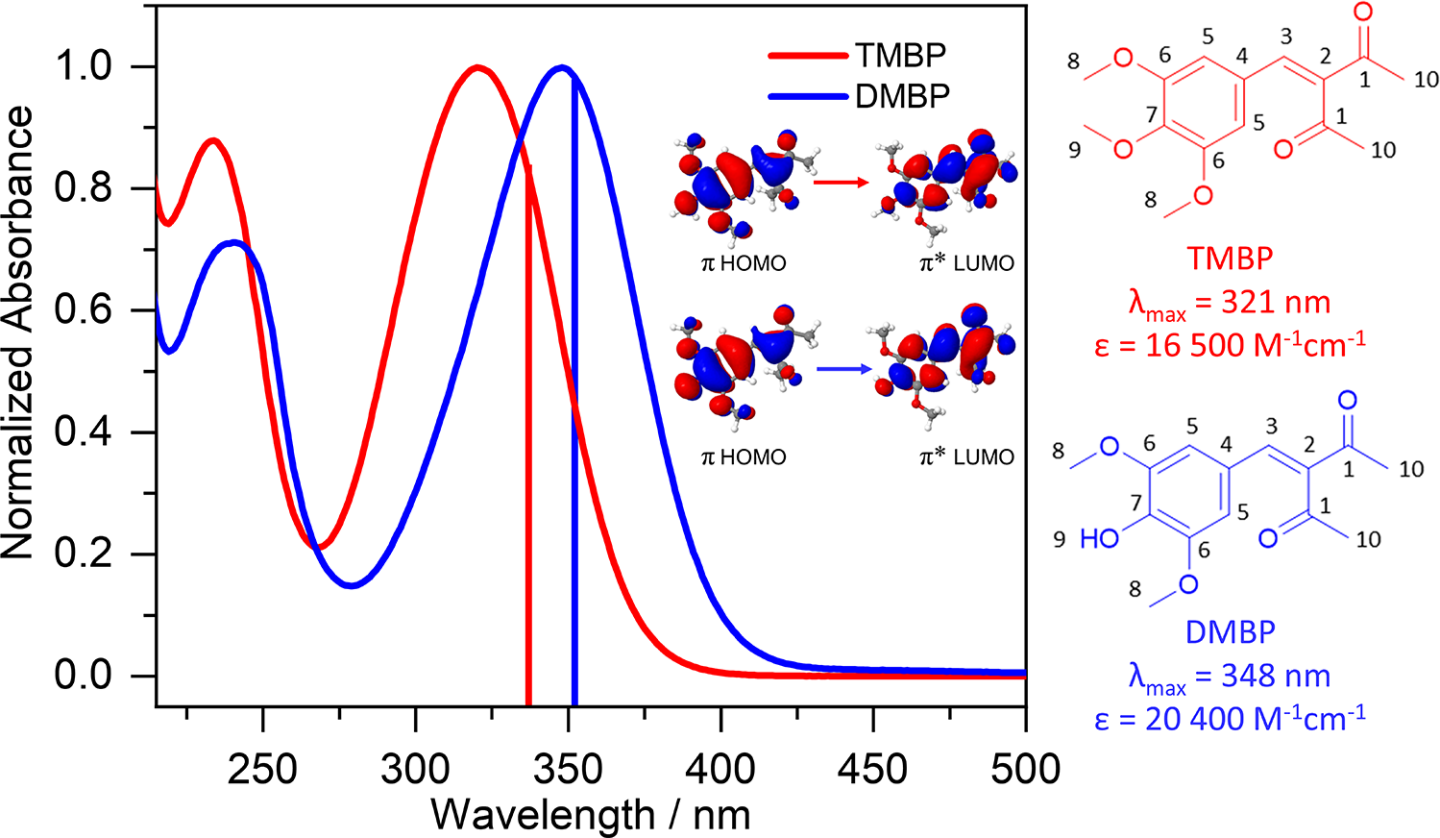
Steady-state UV–visible absorption spectra of TMBP (red) and DMBP (blue) obtained in ethanol. The wavelength of the theoretically predicted strongest transition (corresponding transition orbitals shown inset) for each molecule is presented as a vertical line, with the color matching the corresponding experimental absorption spectrum. Also shown are the structures of TMBP (red) and DMBP (blue), the λ_max_, and the extinction coefficient.

To address these shortcomings, sunscreen scientists are developing and testing new candidate molecules for UVA filters, including those from natural sources such as microbial species and plants.^16−21^ Alternatively, photostabilizers could be added to the formulation of the currently approved UVA filters to improve their photostability. For example, 3-(3,4,5-trimethoxybenzylidene) pentane-2,4-dione (TMBP), with the structure shown in Figure 1, is a UVA absorber designed for use as a photostabilizer and sun protection factor (SPF) booster in sunscreen formulation with the commercial name Synoxyl HSS (sold by Sytheon Ltd.). A previous study of TMBP focused on its steady-state photostability and SPF boosting properties in formulations with UV filters, such as homosalate, octyl salicylate, and avobenzone.^22^ This study showed that TMBP is able to improve the photostability of avobenzone and increase the SPF of the formulation by ∼50%.

Since TMBP absorbs UV radiation with high extinction coefficient (16 500 M^–1^ cm^–1^) in the region where photoprotection is required (UVB–UVA), it stands to reason that this photostabilizer can also serve the dual role as a UV filter. Hence, the objectives of the current study are to unravel the ultrafast photochemistry of TMBP alongside its phenolic derivative, 3-(4-hydroxy-3,5-dimethoxybenzylidene) pentane-2,4-dione (DMBP, see Figure 1), since phenolic substituents are a recognized means of promoting nonradiative decay pathways.^23,24^ Dynamic information obtained from this study could reveal molecular-level insight into the photoprotection mechanisms adopted by TMBP and DMBP, providing guidance into further development of efficient molecules for application as UV filters in sunscreen formulations. We utilize ultrafast transient electronic and vibrational absorption spectroscopy (TEAS and TVAS) to gain insight into the electronically excited state and ground state dynamics in different “solvent” environments: (1) ethanol and (2) caprylic/capric triglyceride (CCT) bulk solution, along with (3) deposition of the CCT bulk solution on a synthetic skin mimic VITRO–CORNEUM (VC), to achieve a close-to-application environment of a sunscreen. VC is a thin film–substrate that mimics the thickness, viscoelasticity, chemical reactivity, and surface properties (hydration and moisturizing) of the human stratum corneum. Furthermore, we expose the separate solutions of TMBP and DMBP to radiation from a solar simulator and record their UV-visible spectra to ascertain their long-term photostability. To complement these studies and better understand their photoprotection pathways, we employ density functional theory (DFT) and time-dependent (TD)DFT computational methods.

## Experimental and Computational Methods

All reagents were purchased from Sigma–Aldrich, TCI, Merck, or VWR and used as received. Solvents were purchased from Thermo Fisher Scientific and VWR. Deuterated dimethyl sulfoxide (DMSO-*d*_*6*_ < 0.02% H_2_O) and acetone (*d*_*6*_ < 0.02% H_2_O) were purchased from Euriso-top. TMBP and DMBP are synthesized according to the previously described procedure.^25^ NMR analyses were recorded on a Bruker Fourier 300 Spectrometer. ^1^H and ^13^C NMR spectra of samples were recorded at 300 and 75 MHz, respectively.

Samples of separate TMBP and DMBP (∼400 μM) were prepared in both ethanol and CCT for long-term photostability studies. The UV-visible measurements were taken in a 1 mm path length quartz cuvette using a Cary 60 Spectrometer (Agilent Technologies) both before irradiation and at various times during 2 h of sunlike irradiation with a solar simulator (Oriel LCS-100) having an irradiation power equivalent to one Sun (∼1000 W/m^2^). In an attempt to investigate the possible formation of any long-lived photoproducts upon UV excitation of both TMBP and DMBP, ^1^H NMR spectra of separate samples prepared to 0.5 M were recorded in deuterated ethanol (ethanol-*d*_*6*_) before and after 5 h of continuous irradiation under a solar simulator (Oriel Instruments, 91191–1000) with irradiance power equivalent to 7 Sun (∼7000 W/m^2^). A solar simulator with a higher irradiance power was used for the NMR studies to increase the chance of forming any potential photoproduct. The infrared (IR) absorption spectra of 30 mM of each solution of TMBP and DMBP in ethanol were taken using an FTIR spectrometer (VERTEX 70v, Bruker). The sample holder is a Harrick cell fitted with two CaF_2_ windows separated by 150-μm polytetrafluoroethylene (PTFE) spacers. The FTIR spectra were recorded over a wavenumber range of 500–4000 cm^–1^ with a resolution of 1 cm^–1^. The emission spectrum and emission lifetime were also recorded for both TMBP and DMBP in ethanol at a concentration of ∼10 μM and 1 cm path length quartz cuvette. Emission spectra were collected using the Horiba FluoroLog-3 with an excitation wavelength at the λ_max_ of each sample and slit width of 2.5 nm. A NanoLED with a central wavelength of 318 nm was used as the excitation source for the emission lifetime measurements, also provided by the FluoroLog-3.

The femtosecond (fs) TEAS setup and procedure used to explore the photodynamics of TMBP and DMBP in solution has been detailed previously,^16,26−29^ and only information specific to the present experiments is reported here. Separate samples of TMBP and DMBP were prepared to 1 mM concentration in ethanol and CCT. In all cases, the pump excitation wavelength was chosen to match the relevant λ_max_. The sample was delivered through a demountable Harrick Scientific flow-through cell equipped with two CaF_2_ windows separated by 250 μm PTFE spacer, thereby defining the optical path length of the sample. The samples were circulated using a diaphragm pump (SIMDOS, KNF) recirculating from a 25 mL sample reservoir, with a maximum pump–probe delay of 2 ns. Similar measurements were repeated for TMBP and DMBP at a concentration of 30 mM to replicate dynamic information at the concentration used for the TVAS measurements (discussed below). To reduce the absorption of the white light continuum (when using 30 mM concentration), which could result in cutting off the informative region of the probe spectrum, a 6 μm PTFE spacer was used to define the optical path length.

For the study on the skin mimic, we have modified our TEAS setup to accommodate a horizontally mounted sample holder alongside the conventional vertical sample holder used in the solution-phase TEAS measurements. A schematic of both setups is presented in Supporting Information (SI) Figure S1. The horizontally mounted sample holder allows transient absorption measurements on thin film solutions without the loss of sample due to the effects of gravity. To achieve this, both pump and probe beams are first periscoped upward after which the probe beam is reflected onto an off axis parabolic (OAP) mirror. This OAP focuses the probe beam to a spot size of ∼50 μm onto the horizontally mounted sample. The probe beam is then collimated by a second OAP located below the sample before it is then sent into a fiber coupled spectrometer (Avantes, AvaspecUSL1650F-USB2). The pump beam is overlapped with the probe beam through the use of a gimble-mounted mirror and is focused to a point beyond the sample, through the use of a 500 mm focal length lens, giving a beam diameter at the sample of ∼350 μm. Samples are prepared in CCT bulk solution at a concentration of 30 mM and deposited on the skin mimic placed on a 2 mm CaF_2_ windows. During the measurements, the sample is translated within the plane of the probe focus, to ensure a fresh sample spot size is irradiated for each spectrum taken. The difference in the concentration of the samples studied in bulk solution (1 mM) vs skin mimic surface (30 mM) is in keeping with the nature of the sample delivery for each measurement. In the bulk solution, sample is delivered with a path length of 250 μm and giving change in optical density (ΔOD) of ∼0.01. Contrarily, in the skin mimic study, samples are thinly applied to the surface with the aid of a fine brush, resulting into smaller sample path length (estimated through UV–visible spectrum to be ∼50 μm); coupled with the nature of the skin surface, this results in low signal-to-noise ratio, hence the need for a high concentration to improve the signal and achieve a ΔOD of ∼0.004.

The TVAS setup employed a similar approach with the TEAS setup and has been reported in detail previously;^16,18^ again, only information specific to the current experiments is reported. Due to the strong IR absorption of CCT in the region of interest, TVAS measurements were taken in ethanol only. To note, the TEAS measurements in ethanol show commensurate dynamics to those in CCT environment. 30 mM concentrations of TMBP and DMBP were prepared for TVAS measurement, using the same sample delivery system as for the TEAS studies, but with a 150 μm thick spacer and a maximum pump–probe delay of 1 ns. The concentration was increased to 30 mM to achieve a workable signal with ΔOD of ∼0.001. In both TMBP and DMBP, the UV pump pulse employed for photoexcitation corresponds to their respective λ_max_, with a mid-IR probe pulse centered at 1580 and 1590 cm^–1^ respectively, to match their IR peak absorption.

For the electronic structure calculations, the structures of TMBP and DMBP were generated using visual molecular dynamics (VMD) with the molefacture plugin.^30^ The geometries of the ground state (S_0_) of both molecules were optimized with DFT using NWChem software.^31^ Optimizations were carried out using a 6-311++G** basis set with the PBE0 functional.^32^ Furthermore, DFT frequency calculations were carried out at the 6-311++G**/PBE0 level of theory on the S_0_ optimized geometries for both TMBP and DMBP to obtain wavenumbers to help guide the assignment of the ground state vibrational modes contributing to the measured FTIR spectra. Using the same basis set and functional reported above, the vertical excitations of the optimized S_0_ structures were computed with TD-DFT. The Franck–Condon (FC) geometry for each molecule was further optimized in its respective excited state using TDDFT until it converged into a minimum. Optimization of the TMBP geometry in the initially populated S_2_ state revealed rapid internal conversion (IC) to the S_1_ state at a point close to the S_2_ FC geometry. The internally converted S_1_ geometry was then further optimized in the S_1_ state until it converged into a minimum. Contrarily, DMBP initially populates the S_1_ state and during optimization, it relaxes into a minimum in the same state. In all the calculations, the conductor-like screening model (COSMO) was used to model the effect of solvent (ethanol) dielectric parameters.^33,34^ The default COSMO solvent model for ethanol within NWChem was used, descriptors of which are based on the Minnesota Solvent Descriptor Database.^35^

## Results and Discussion

The synthesized TMBP and DMBP were fully characterized by ^1^H and ^13^C NMR spectroscopy, as well as high-resolution mass spectrometry; further details can be found in the SI Figure S2–S7. The experimental UV–visible spectra of both TMBP (λ_max_ = 321 nm) and DMBP (λ_max_ = 348 nm) in ethanol solution are shown in Figure 1. Additional UV–visible spectra in CCT for both molecules are reported and discussed later.

### TMBP and DMBP Structures in the Ground and Excited Electronic States

Using DFT, we optimized the geometry of each molecule in the ground electronic state and calculated the vertical excitation energy with TD-DFT. The optimized S_0_ structure from our calculation is shown in Figure S8 of the SI. For both TMBP and DMBP, the S_0_ has a close-to-planar geometry, with one of the ketone groups out of plane by 67.9° (for TMBP) and 72.7° (for DMBP). In addition to one of the ketone groups, the methoxy group at the *para* position of the benzene for TMBP is also perpendicular to the plane for the S_0_ optimized geometry (Figure S8 of the SI). The calculated orbital character in a vacuum reported by Chaudhuri et al.^22^ for TMBP showed that the S_1_ ← S_0_ transition assigned to the λ_max_ absorption corresponds to nπ*. In contrast, the vertical excitation in implicit ethanol environment in the current study indicates that the absorption band at the λ_max_ corresponds to S_2_ ← S_0_ and S_1_ ← S_0_ transition in TMBP and DMBP respectively, both having ππ* character (see the Figure S9 for computed molecular orbitals) and in line with previous literature on similar symmetrically substituted molecules.^16,24^ The difference between the two results above might be attributed to the variation in molecule environment. As mentioned earlier, in TMBP, vertical excitation initially populates the S_2_ state, the assignment of this transition is due to the greater oscillator strength compared to S_1_ ← S_0_ (see Table S1). For DMBP, vertical excitation populates the S_1_ state. Excited state geometry optimizations were performed for TMBP and DMBP to determine the energies of their S_1_ states after structural relaxation from the FC region. The optimization of both TMBP and DMBP geometry in the excited state converges to a minimum on the S_1_ state having an increasing C_3_=C_2_ bond length and twist around the allylic double bond (see Figure S8 of the SI). Additionally, the *para*-methoxy group on the phenyl ring of TMBP rotates to an in-plane position with the benzene ring. The orbital character for excitation transitions of TMBP and DMBP are shown in the SI, Figure S9. We note here that we have not fully characterized the potential energy surface (PES) of TMBP and DMBP in the excited state; however, to assign the dynamical processes to our experimental data, the calculations reported above were compared with PES calculations of similar molecules reported previously.^16,24^

Furthermore, the excited molecular orbital (MO) in the optimized S_1_ geometry for both molecules is located mostly on the diketone group (Figure S10), demonstrating charge transfer (CT) character for the molecules as they relax along the S_1_ potential energy surface. The dynamics from the FC region of the initially excited molecules in both cases (TMBP and DMBP) are therefore expected to involve twisted intramolecular charge transfer (TICT). Again, this is in line with previously studied, and similar, symmetrically substituted systems.^16,24^ These form the basis for the interpretation of the ultrafast transient absorption spectroscopy data reported herein.

### Transient Electronic Absorption Spectroscopy of TMBP and DMBP

**Figure 2:**
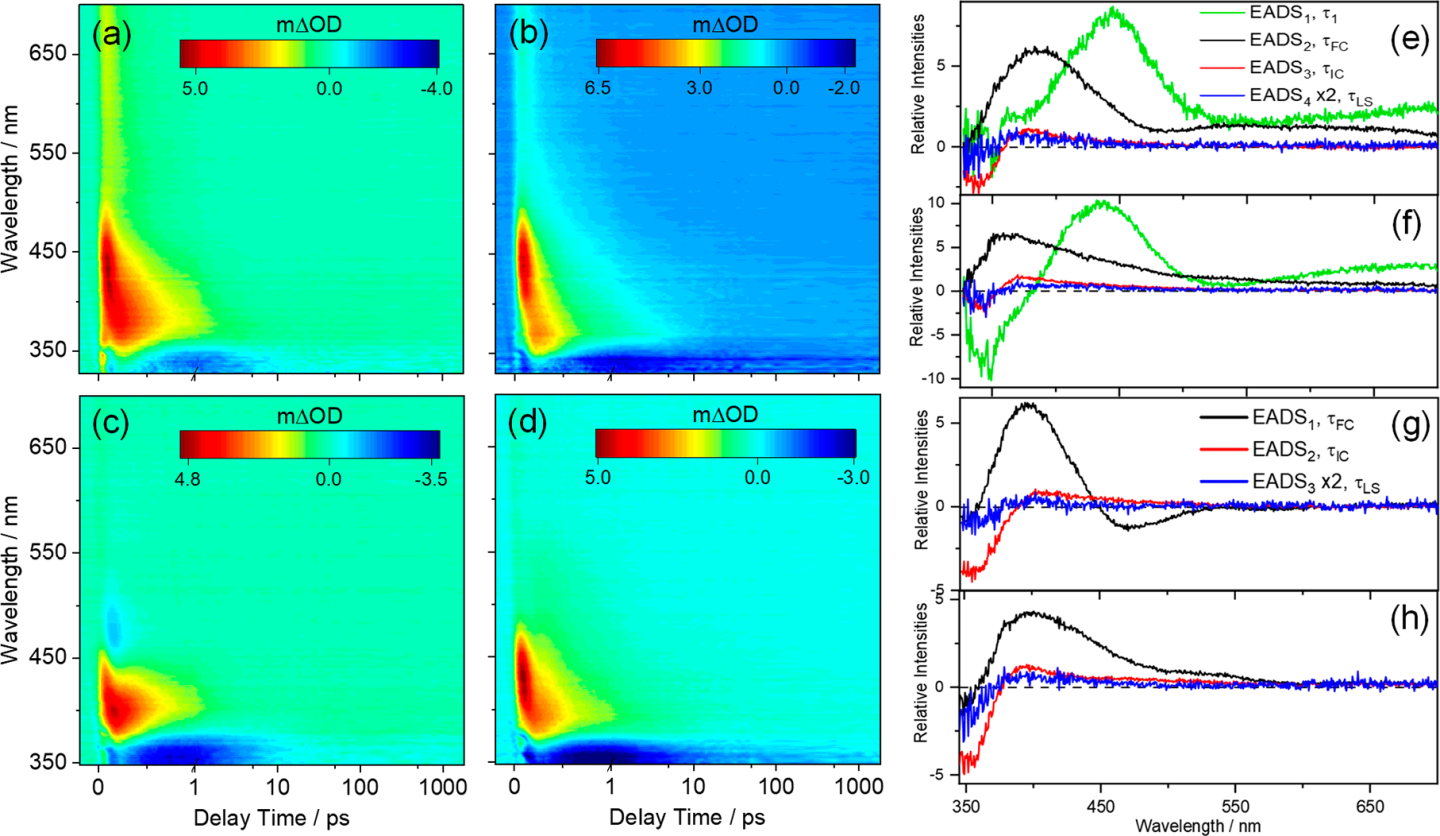
TEA spectra presented as a false color heatmap for 1 mM TMBP in (a) ethanol and (b) CCT and for 1 mM DMBP in (c) ethanol and (d) CCT, following photoexcitation at their respective λ_max_. In all cases, the pump–probe delay time is presented on a linear scale until 1 ps and then as a logarithmic scale between 1 and 2000 ps. The evolution associated difference spectra (EADS) produced by the fitting procedure are shown in panels e and f for TMBP in ethanol and CCT respectively, and in panels g and h for DMBP in ethanol and CCT, respectively. EADS_4_ in panels e and f, together with EADS_3_ in panels g and h, are all multiplied by two as a visual aid.

The transient electronic absorption (TEA) spectra of TMBP and DMBP following photoexcitation at their respective λ_max_, both in ethanol and CCT at 1 mM are reported as false color heat maps in Figure 2a–d; the same data are reported as line plots in Figure S11. Additional TEA spectra obtained for separate solutions of TMBP and DMBP in ethanol at 30 mM, and in CCT at 30 mM deposited on VC (denoted TMBP VC/CCT and DMBP VC/CCT, henceforth) are reported in Figures S12 and S13 of the SI, respectively. Given the evident similarities between the TEA spectra measured when exciting both TMBP and DMBP at the appropriate λ_max_ values, in all the solvent environments, i.e., ethanol, CCT and VC/CCT, the results are discussed as a collective.

The spectra showed two distinct features in all cases. The first is a negative feature located at the edge of the shortest wavelength region of our probe spectrum, i.e., ∼330 nm in TMBP and ∼350 nm in DMBP. Comparison with the UV-visible spectra reported in Figure 1 implies that this feature is attributable to the ground state bleach (GSB). The second is an intense positive feature peaking at ∼440 and ∼410 nm for TMBP and DMBP respectively, in both solvents and assigned to excited state absorption (ESA). For TMBP, this feature extends toward the red end (∼700 nm) of the TEA spectrum at early time delays. The presence of this feature from time zero (Δ*t* = 0, i.e., where our pump and probe beams temporally overlap) is an indication that the feature corresponds to the absorption of excited state population from the FC region in both molecules. Also, the blue shifting of the ESA with increasing delay times (Δ*t* > 0, more evident in the EADS reported in Figure 2e–h) is a signature of absorption of vibrationally hot molecules from the S_0_ state following IC from S_1_.^36−38^ Hence, it is plausible that this feature is a convolution of the two processes highlighted above. Furthermore, in all cases, this feature rapidly decays to about zero within pump–probe delays of ∼5 ps for TMBP and ∼2 ps for DMBP in both solvents. On closer inspection, in addition to the two features described above, a weak negative feature centered at ∼480 nm is observed in the TEA spectra of DMBP in ethanol. This feature is assigned to stimulated emission (SE). The presence of SE in DMBP dissolved in ethanol but not CCT is likely an effect of solute–solvent interaction with the polar solvent. This solvent dependent feature has been reported for sinapate esters, a group of similar molecules to TMBP and DMBP, previously.^39,40^ Furthermore, the presence of SE at early Δ*t* and its decay at delay times <500 fs indicates that this feature is likely derived from the photoexcited DMBP in the S_1_ state FC (or nearby) region, and its decay is a measure of the relaxation of the electronic excited state dynamics.

To extract the excited state kinetic information from the TEA spectra of each molecule, a global sequential ) decay model implemented through the Glotaran software package was employed.^41,42^ The extracted time constants are reported in Table 1. The quoted errors in Table 1 are those returned by the fitting software to twice the standard error, though the quality of the fits is better evaluated by inspecting the associated residuals reported in SI Figure S14. Where the error returned by the fitting package was shorter than the instrument response time, the error is quoted as half the instrument response (as determined via the solvent-only transients presented in Figure S15).

**Table 1:** Summary of the Time Constants and Associated Errors Extracted from Data Collected for TMBP and DMBP in Solution of Ethanol, CCT, and VC/CCT with TEAS

sample		ethanol (1 mM)	ethanol (30 mM)	CCT (1 mM)	VC/CCT (30 mM)
TMBP	τ_1_ (fs)	100 ± 40	110 ± 40	within IRF	within IRF
	τ_FC_ (fs)	400 ± 40	290 ± 40	320 ± 40	340 ± 40
	τ_IC_ (ps)	3.5 ± 0.1	2.4 ± 0.1	5.2 ± 0.2	5.8 ± 0.3
	τ_LS_ (ns)	>2	>2	>2	>2
DMBP	τ_FC_ (fs)	380 ± 40	380 ± 40	380 ± 50	350 ± 50
	τ_IC_ (ps)	2.2 ± 0.1	2.2 ± 0.1	3.2 ± 0.1	3.7 ± 0.2
	τ_LS_ (ns)	>2	>2	>2	>2

The first dynamical process extracted from the fit of TMBP, defined by τ_1_, occurs with a relatively short time constant (60–110 fs) that is mostly within the instrument response function (IRF) in all the solvent environments. However, based on our electronic structure calculations (discussed earlier), the photoexcitation of TMBP proceeds by S_2_ ← S_0_ which undergoes rapid IC to the S_1_ state with minimal geometry distortion. Following IC, TMBP further relaxes along the S_1_ potential energy surface. Hence, we speculate that IC from S_2_ → S_1_ happens within the IRF, but we are unable to accurately assign a lifetime to this process. In both TMBP and DMBP, τ_FC_ which ranges from 320–400 fs is assigned to the relaxation of the FC geometry molecule along the S_1_ reaction coordinate toward the S_1_/S_0_ conical intersection (CI). This relaxation is accompanied by a geometry change, i.e., a twisted geometry around the C_3_=C_2_ allylic bond (see Figure 1 for atom number) with charge transfer character, referred to as twisted intramolecular charge transfer (S_1-TICT_) henceforth. The τ_FC_ for both TMBP and DMBP agree with the time constant reported for TICT dynamical process in our previous work on similar symmetrically substituted systems.^16,24^ It is worth noting that the presence of SE in the EADS_1_ for DMBP in ethanol, corresponding to τ_FC_ in Figure 2g is an indication that within this time constant, the photoexcited population is in the electronic excited state.

The time constant denoted by τ_IC_ in both TMBP and DMBP, is assigned to the decay of the S_1-TICT_ excited state population through IC, which is mediated by the S_1_/S_0_ CI. Confirmation for this assignment is largely drawn from the TVAS data (discussed later) and previous literature on similarly symmetric substituted molecules.^36−38^ Furthermore, it is expected that following IC of excited state population, vibrational hot S_0_ molecules will be formed, which will eventually transfer the excess energy via vibrational energy transfer both intramolecularly and intermolecularly (to the solvent environment), collectively termed vibrational cooling. The absorption of this vibrationally hot S_0_ species following IC is usually observed on the red wavelength side of the thermalized S_1_ ← S_0_ band (i.e., in the 350–500 nm range in the case of TMBP and 370–450 nm in DMBP). This absorption would then progressively narrow and blue-shift at a longer delay times. While this absorption and its blue shifting is observed in the TEA spectra of TMBP and DMBP in all solvents, our global fit does not return any specific time constant for this process. This is likely due to the overlapping of absorption bands (discussed earlier). Nonetheless, the τ_IC_ values extracted from data measured in ethanol for both molecules are consistently shorter than the corresponding quantities measured in CCT and VC/CCT. This is a further indication that the overlapping of the absorption band of the vibrationally hot S_0_ species with the ESA band might contribute to the time constant extracted for τ_IC_, since vibrational energy transfer will be faster in a strongly interacting (polar) solvent.^43^ As an aside, we note the absence of SE in the EADS_2_ corresponding to τ_IC_ in Figure 2g and the appearance of ESA in the SE region that sensibly corresponds with a hot electronic ground state. As a mild caution, the absence of the SE feature in the EADS_2_ could also be attributed to larger ESA band overlapping with the weaker SE. Also, we note the variation in τ_IC_ for TMBP when dissolved in ethanol at different concentration (i.e., 1 mM vs 30 mM, see Table 1); in contrast, for DMBP, τ_IC_ remained the same at both concentrations. At present the origin of the variation observed in TMBP is unclear. The TVAS data (discussed later) provide a clearer picture of the excited state population relaxation and the vibrational energy transfer dynamics.

The final time constant, τ_LS_ > 2 ns, for both TMBP and DMBP corresponds to the dynamics of the incomplete recovery of the GSB, and a mild ESA centered ∼400 nm in Figure 2e–h, and in the TAS at 2 ns shown in Figure S16. We recognized that the origin of this long-lived feature could be attributed to multiple sources including any trapped population in the excited state, which could be either the S_1_ or a triplet state. Alternatively, this long-lived species could also be assigned to a potential molecular photoproduct. The extracted emission lifetime of ∼5 ns (both aerated and nitrogen-flushed samples, see Figure S17 of the SI) along with the small Stokes shift in the emission spectrum of both TMBP and DMBP (Figure S18) suggests that the emission is from the singlet state which decays via fluorescence. A clue to this comes from our time-resolved fluorescence studies. We add that a previous study of TMBP (in propanol) at 77 K observed phosphorescence.^22^ Given our experimental limitation, we are unable to confirm phosphorescence in ethanol and, thus, cannot rule out the possibility of intersystem crossing here.

### Transient Vibrational Absorption Spectroscopy of TMBP and DMBP

**Figure 3:**
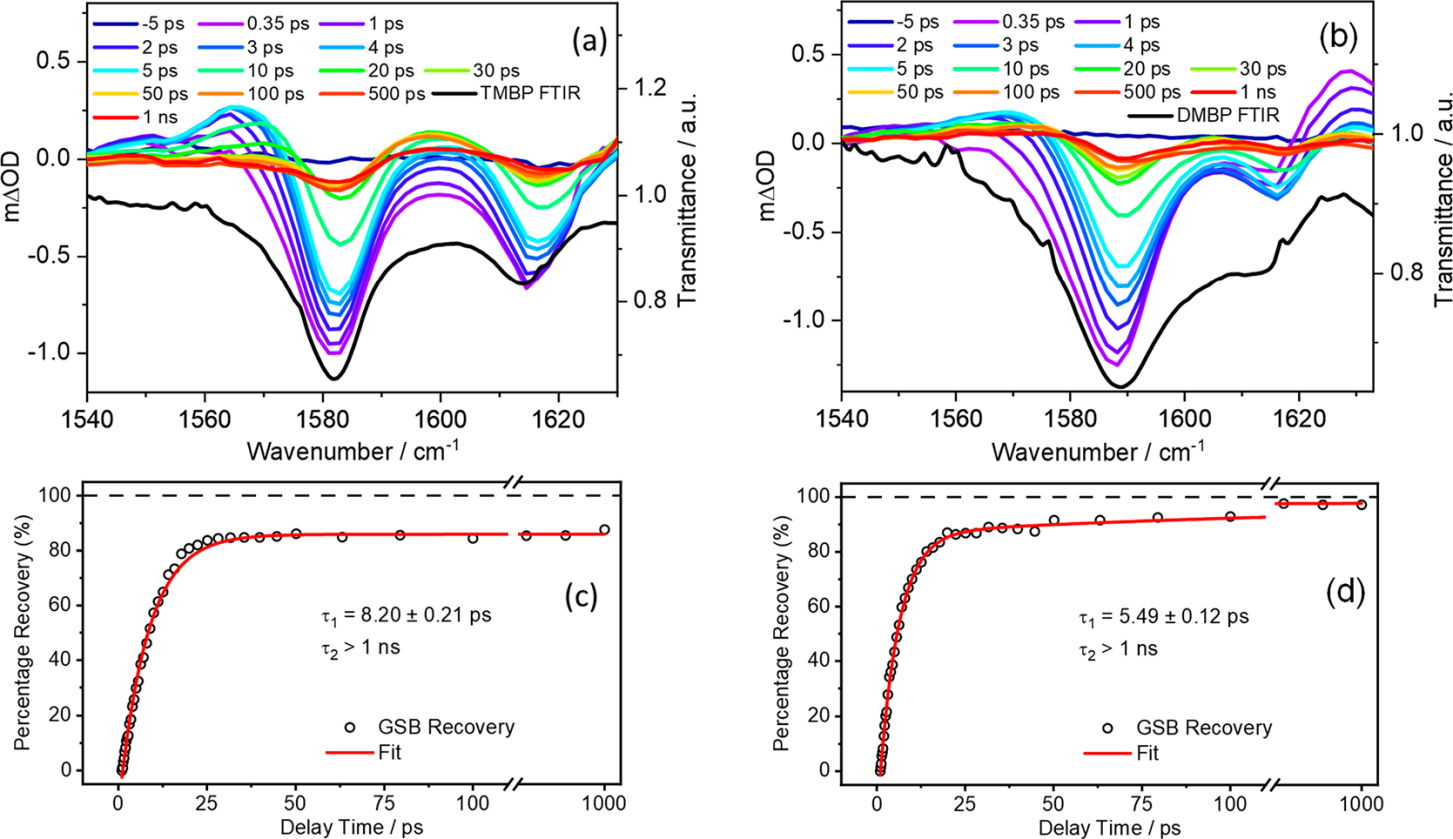
TVA spectra obtained for 30 mM ethanolic solutions of (a) TMBP and (b) DMBP, both photoexcited at their respective λ_max_ and using a broadband IR probe pulse centered at 1580 cm^–1^ for TMBP and 1590 cm^–1^ for DMBP. Both spectra are presented as smoothed colored line plots of mΔOD (left-hand *y*-axis) vs probe wavenumber at selected pump–probe delay times. The steady-state FTIR spectra are shown as black lines in the respective panels, with the transmittance scale shown on the right-hand *y*-axis. The kinetics of (c) TMBP and (d) DMBP GSB recovery for the prominent vibrational bands (raw data as open circles and fit as solid red line) centered at ∼1580 cm^–1^ for TMBP and ∼1590 cm^–1^ for DMBP are also reported. In both TVA spectra, the data were fitted with biexponential functions with the delay times plotted linearly until 110 ps; then, there is a break until 600 ps beyond which the 600–1000 ps data are plotted on a logarithmic scale to show incomplete GSB recovery.

Complementary TVAS measurements, i.e., UV-pump/IR-probe, carried out to monitor the electronic nonadiabatic dynamics of the S_1_ → S_0_ relaxation, are reported in Figure 3 for both TMBP and DMBP.

We begin by discussing the steady-state FTIR spectra of TMBP and DMBP in ethanol (see Figure S19) and the transient vibrational absorption (TVA) spectra. On the basis of the FTIR spectra, the negative vibrational bands centered at ∼1580 cm^–1^ and ∼1590 cm^–1^ in TMBP and DMBP, respectively, and at 1615 cm^–1^ in both TVA spectra are assigned as GSB arising from the photoexcitation of the S_0_ molecules. Frequency calculations suggest that these vibrational bands are assigned to the allylic C=C stretching at ∼1580 cm^–1^ and aromatic C–H bending together with C=C stretching at ∼1605 cm^–1^ in both TMBP and DMBP (see Table S2). In the TVA spectra, the GSB begins to recover as the delay time increases, an indication of relaxation of the photoexcited molecule back to the S_0_. The noticeable GSB features remaining after the limiting 1 ns delay time of our measurements in both TMBP and DMBP is an indication that certain population of the photoexcited molecules do not, or are yet to return, back to the ground state. Also, at 1 ns, all the positive absorption bands have completely decayed. Quantitative evaluation of the GSB recovery revealed that 88% (95%) of the photoexcited TMBP (DMBP) returned back to their respective ground state after 1 ns. This is in line with our TEAS measurements, indicating the presence of a small long-lived, dynamical component. To the left of the GSB feature in the TVA spectra of both TMBP and DMBP is a positive feature which is centered at ∼1565 cm^–1^ and rises to maximum in about 5 ps before it starts to decay. This feature can be sensibly assigned to the absorption of the vibrational hot molecules in S_0_ state formed following IC from the S_1_. Further support to this assignment comes from the TVA spectra of DMBP. Here we see a second positive feature centered at ∼1630 cm^–1^. This feature is present at the instant of maximum intensity of the GSB signal, i.e., at time delay of 0.35 ps, indicating that it originated from the excited state; as this feature decays, we see a growth in the vibrationally hot S_0_ molecules. We assign the ∼1630 cm^–1^ feature to ESA.

The kinetic information from the TVA spectra was extracted as follows. A 5 cm^–1^ integration window was applied to each spectral feature in the TVA spectra (GSB and ESA). The intensity changes to these features were then fit to biexponential (GSB) and monoexponential (ESA band) decay functions. The extracted time constants for the GSB are shown in Figure 3c and 3d. A global fitting procedure has not been employed in these fittings as the key interest is just the recovery of S_0_ population (as revealed by the probed ground state vibrational mode) and decay of the ESA band in DMBP. Furthermore, the exponential fit is started at the instant of maximal GSB signal intensity, i.e., at a pump–probe delay of 0.35 ps in both cases, thereby avoiding any coherent artifacts at early time delays and (or) solvent heating. The recovery of the GSB in TVAS measurements is obtained from the repopulation of S_0_ molecules in their lowest vibrational level, hence the recovery time constant in TVA spectra in this case will be dominated by vibrational cooling in the S_0_ state, as with previously described systems.^44−46^ The extracted time constant of τ_1_ = 8.20 ± 0.21 ps (5.49 ± 0.12 ps) for the GSB recovery of TMBP (DMBP) could therefore be assigned to vibrational cooling in the S_0_ molecules. To gain additional insight into the dynamical processes assigned to the relaxation of TMBP and DMBP, we have fit the absorption bands corresponding to the ESA centered at ∼1630 cm^–1^ in DMBP. This fit returned a time constant of 1.97 ± 0.13 ps indicating that the excited state populations decay within this time constant. The agreement (within error) between this time constant (1.97 ± 0.13 ps) and the τ_IC_ of 2.2 ± 0.1 ps extracted from the TEA spectra of DMBP coupled with the longer time constant for the GSB recovery supports the claim that τ_IC_ in the TEAS measurements corresponds to excited state relaxation and not vibrational cooling.

The extracted τ_2_ in the GSB recovery fit (Figure 3c and 3d) with a time constant longer than 1 ns corresponds to the remaining population (10% in TMBP and 5% in DMBP) which is yet to recover back to ground state. The absence of photoproduct absorption band in the TVA spectra and the emission measurements (discussed earlier) confirm that this long-lived component is most likely a consequence trapped population in the singlet excited state which would decay with a longer time constant via fluorescence. Alternatively, we could be populating an intermediate state that persists beyond the time-window (1 ns) of our experiment.

### Long-Term Steady-State Irradiation of TMBP and DMBP

**Figure 4:**
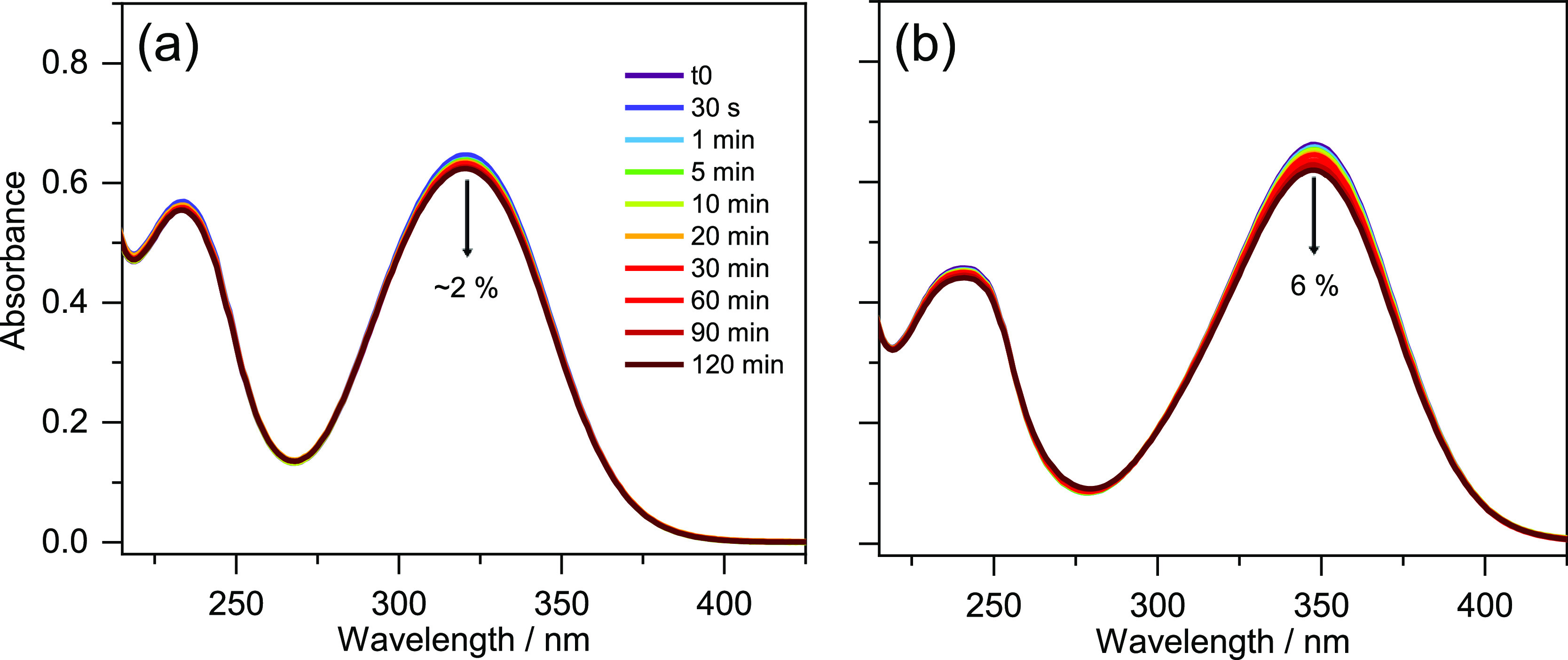
UV–visible spectra before and at various times during 2 h of irradiation with solar simulator in a 1 mm cuvette for (a) TMBP and (b) DMBP in ethanol. The downward arrows denote the observed percentage decrease in λ_max_ absorbance over 2 h of irradiation.

The origin of the incomplete recovery of the photoexcited molecules as observed in both TEAS and TVAS measurements is further explored by steady-state long-term irradiation measurements. This allows us to determine if any photoproducts, formed via photodegradation, could originate from the long-lived species in TEAS and TVAS measurements. As shown in Figure 4, both samples demonstrated high photostability in ethanol, with only small reduction (∼2% for TMBP and 6% for DMBP) observed in sample absorbance at λ_max_ over 2 h of sunlike irradiation. Photostability data obtained in CCT and reported in the SI Figure S20 revealed that TMBP maintained its high photostability with ∼2% reduction in sample absorbance; in contrast, a significant reduction (∼30%) in the absorbance of DMBP is observed. Possible sources of the reduction in absorption including formation of potential photoproduct, photoacid in excited state or reaction resulting from photoexcited CCT are discussed in the SI.

Comparison of the difference spectra between before and after irradiated samples (Figure S21) and the 2 ns TEA spectra (Figure S16) for both TMBP and DMBP in all solvent environment shows that the two spectra only correlate in the region around 350 nm. The ESA feature around 400 nm is not observed in the steady-state difference spectrum. This is an indication that the incomplete recovery of the GSB in both TEAS and TVAS is likely not dominated by photoproducts, but a trapped population in the singlet excited state. Furthermore, the ^1^H NMR spectra obtained before and after irradiation for both samples (shown in Figure S22) indicate no observable difference, implying little or no photoproducts formation. Although the work by Chaudhuri et al.^22^ suggested the formation of photoproducts for TMBP in methanol following steady-state irradiation, such photoproducts are not observable in all our experiments. As a caveat, we note that the photoproduct suggested by Chaudhuri et al.^22^ may have similar proton shift to the starting molecules in ^1^H NMR spectra.

## Conclusion

In conclusion, combining TEAS, TVAS, electronic structure calculations, and steady-state studies, we have provided insights into the excited state photodynamics of TMBP and DMBP in different environmental conditions: in ethanol, an industrial grade emollient, and on skin mimic surface, the latter providing more than just a conventional solvent-chromophore interaction. The ultrafast dynamical studies demonstrate that, after photoexcitation at their respective λ_max_, both TMBP and DMBP return to the ground state on an ultrafast (picosecond) time scale with 88% and 95% relaxation efficiency, respectively. The relaxation mechanism (in either bulk ethanol, CCT, or deposited on a skin mimic) has been determined to be initiated by excited state geometry distortion involving an S_1-TICT_, which takes the excited population out of the Franck–Condon region and toward the S_1_/S_0_ CI. This assignment is supported by previous work on similar systems and our electronic structure calculations. This initial relaxation process is followed by IC of the excited state population to the ground state via the S_1_/S_0_ CI and subsequent vibrational cooling in the S_0_. Although our global fitting of the TEA spectra is unable to return the time constant for vibrational cooling that follows IC, the TVA data and its fit provides information about the presence of this process. Finally, we note a mild incomplete ground state recovery at 2 ns, which we assigned to excited state population trapped in the singlet state. Steady-state studies revealed that these molecules, with the exception of DMBP in CCT, are highly photostable over 2 h of sunlike irradiation with no observable photoproduct. Since sunscreen formulations are prepared in range of solvents including emollients, the photoinstability of DMBP in CCT suggests it might not be suitable for use as UV filter booster or photostabilizer specifically when CCT is involved in the formulation.

Our present studies have also demonstrated the importance of employing a multipronged approach combining TEAS and TVAS with computational methods in understanding the ultrafast relaxation pathways of TMBP and DMBP for use in sunscreen formulation; the ultrafast dynamics of both molecules are similar in all environments, but their stability evidently differs under prolonged exposure to sunlike irradiation. The absorption of both UVB and UVA wavelengths, and dissipation of the absorbed energy nonradiatively on ultrafast time scale revealed that the studied photostabilizers can function as UV filters in sunscreen formulations, thereby serving a dual purpose in a formulation. To finally add: the demonstration that the studied photostabilizers can serve as UV filters also offers promising avenues for application where photon-to-molecule heat generation is crucially required.

## Notes

The data sets presented in this study can be found in online repositories. The names of the repository/repositories and accession number(s) can be found below: Zenodo repository DOI: 10.5281/zenodo.6793991

## Supporting Information Available

TEAS vertical and horizontal mounted sample experimental setup, ^1^H NMR and ^13^C NMR of TMBP and DMBP molecules, mass spectrometry spectra, optimized geometries, and molecular orbitals of TMBP and DMBP, kinetics fit of TEAS and TVAS, TEAS additional data, TEAS fitting residual spectra, solvent alone instrument response, 2 ns TAS of TMBP and DMBP, emission lifetime and emission spectrum, FTIR spectra of TMBP and DMBP in ethanol, UV difference spectrum, and ^1^H NMR before and after irradiation (PDF)
