# Supplementary material for: Insight into the Photodynamics of Photostabilizer Molecules
Source: J Phys Chem A. 2022 Nov 2;126(45):8388–97. doi: 10.1021/acs.jpca.2c05580 (PMC9677431; doi:10.1021/acs.jpca.2c05580)
Supplement: Supplementary file 1 — jp2c05580_si_001.pdf [file jp2c05580_si_001.pdf]

Supporting Information

Insight into the Photodynamics of Photostabilizer Molecules

Temitope T. Abiola,[†] *Benjamin Rioux,*[§] *Sharanjit Johal,*[†] *Matthieu M. Mention,*[§] *Fanny Brunissen,*[§] *Jack M. Woolley,*[†] *Florent Allais*[§] *and Vasilios G. Stavros*[†*]

† Department of Chemistry, University of Warwick, Gibbet Hill Road, Coventry, CV4 7AL

§ URD Agro-Biotechnologies Industrielles (ABI), CEBB, AgroParis Tech, 51110, Pomacle, France

CORRESPONDING AUTHOR

* v.stavros@warwick.ac.uk

Table of Contents

A) SCHEMATIC OF THE TEAS SETUP EMPLOYED 3

B) CHARACTERIZATION OF TMBP AND DMBP 4

C) COMPUTATIONAL RESULTS 8

D) KINETIC FITS 10

i) TEAS 10

ii) TVAS 11

E) TEAS ADDITIONAL DATA. 11

F) RESIDUALS FOR THE FITS OF TMBP AND DMBP IN THEIR DIFFERENT SOLVENT ENVIRONMENTS 13

G) SOLVENT ALONE INSTRUMENT RESPONSE 13

H) 2 NS TRANSIENTS FOR TMBP AND DMBP IN THEIR DIFFERENT SOLVENT ENVIRONMENT 14

I) FLUORESCENCE LIFETIME AND EMISSION SPECTRA MEASUREMENTS 15

J) FTIR SPECTRA 16

K) STEADY-STATE PHOTOSTABILITY OF TMBP AND DMBP IN CCT. 16

L) UV/VISIBLE DIFFERENCE SPECTRUM. 17

M) ^{1}H NMR OF TMBP AND DMBP BEFORE AND AFTER SOLAR IRRADIATION 18

N) SUPPLEMENTARY REFERENCES 18

A) Schematic of the TEAS setup employed

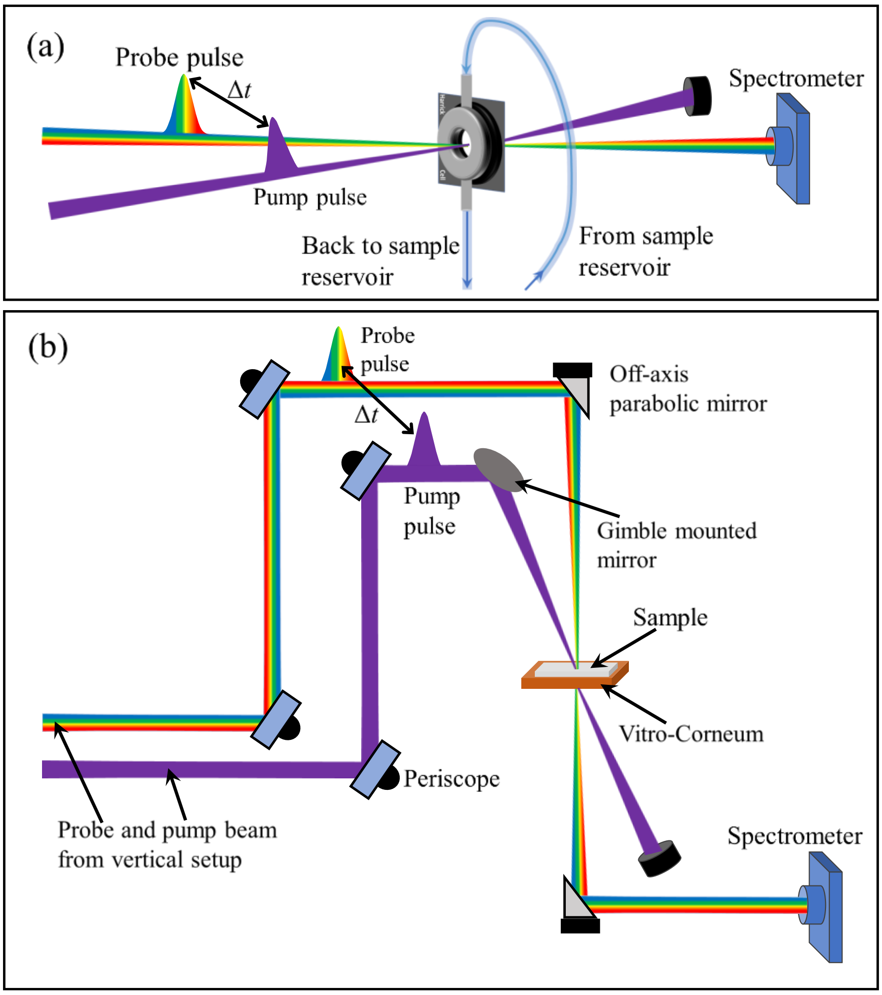

Figure S1. Schematic of the TEAS setup used for the reported transient electronic absorption experimental data when (a) sample is mounted vertically, and (b) when sample is mounted horizontally.

B) Characterization of TMBP and DMBP

TMBP: yield: 31%. ^{1}H NMR (300 MHz, 25 °C, $(CD_3)_2CO$): δ (ppm) = 7.60 (1H, s, H-3), 6.83 (2H, s, H-5), 3.82 (6H, s, H-8), 3.78 (3H, s, H-9), 2.40 and 2.29 (6H, 2s, H-10). ^{13}C NMR (75 MHz, 25 °C, $(CD_3)_2CO$): δ (ppm) = 196.6 (C-1), 153.5 (C-6), 142.2 (C-7), 140.4 (C-2), 139.4 (C-3), 128.6 (C-4), 107.5 (C-5), 59.8 (C-9), 55.5 (C-8), 31.0 and 25.4 (C-10). Melting point: 61-63 ± 0.1 °C. TOF MS ES+: $[M+H]^+$ for $C_{15}H_{19}O_5$: *m/z* 279.1232; found: *m/z* 279.1230.

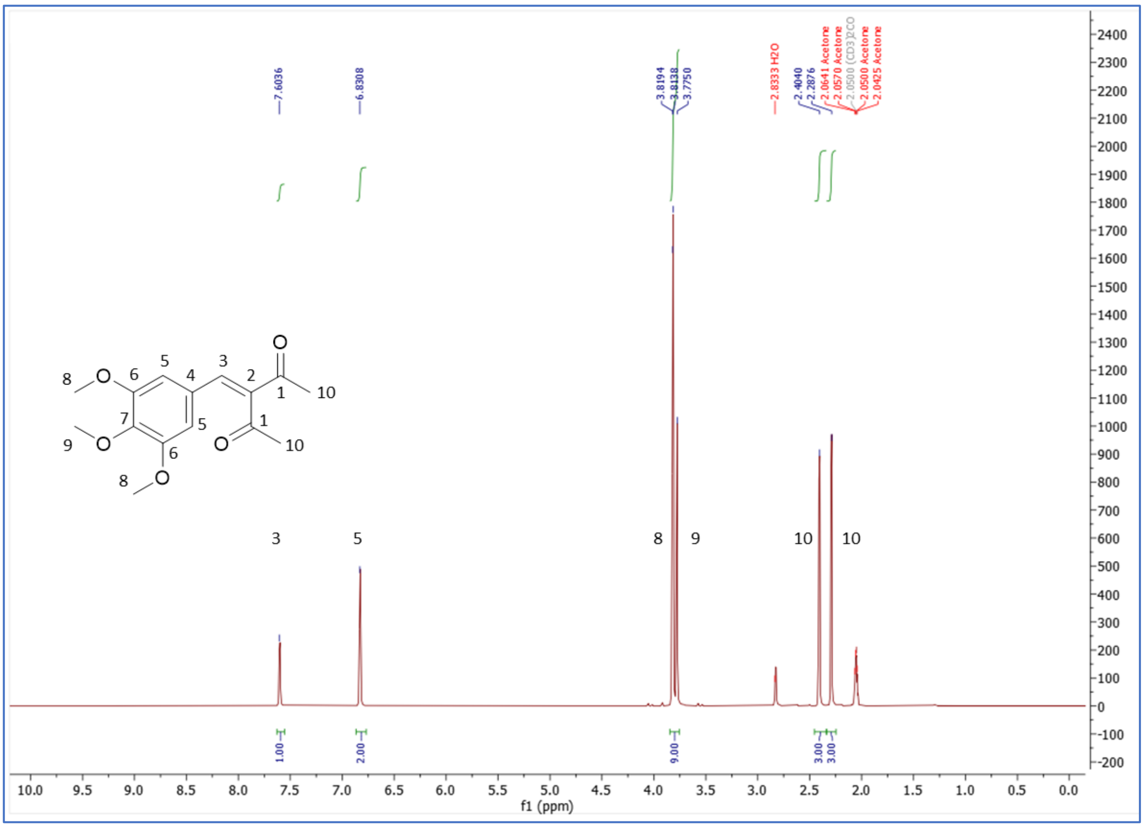

Figure S2. ^{1}H NMR spectrum of TMBP

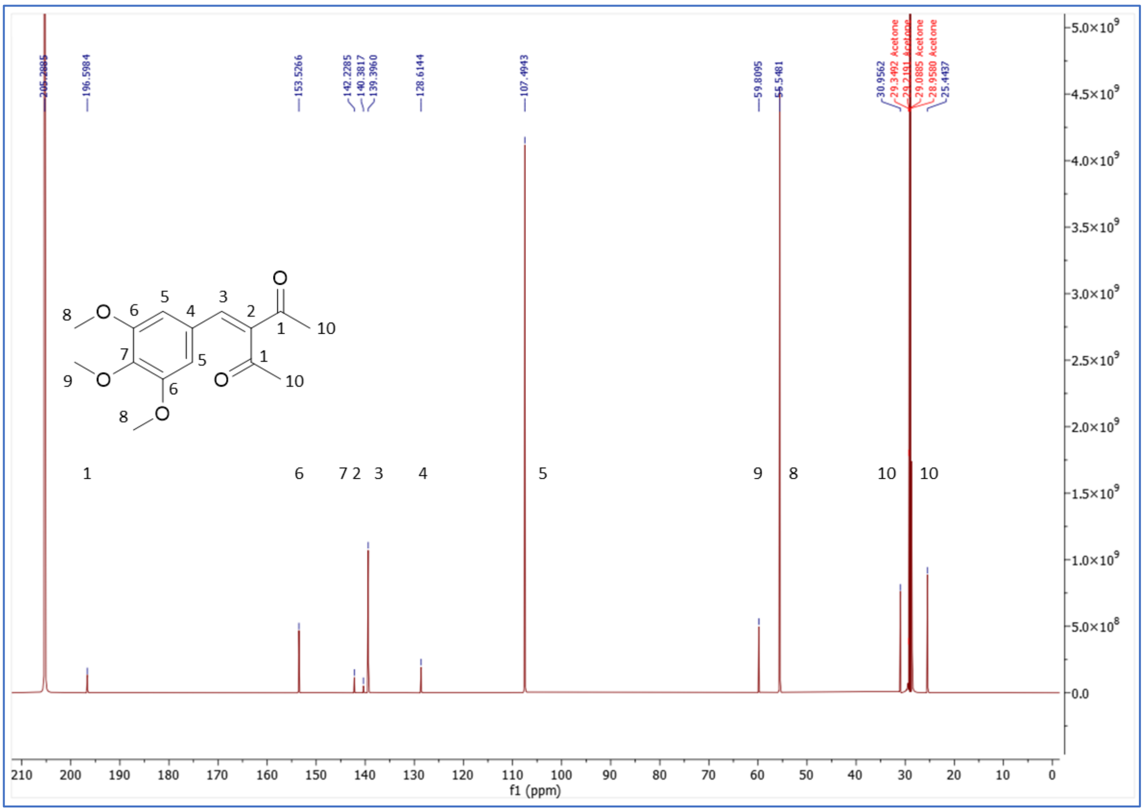

Figure S3. ^{13}C NMR spectrum of TMBP

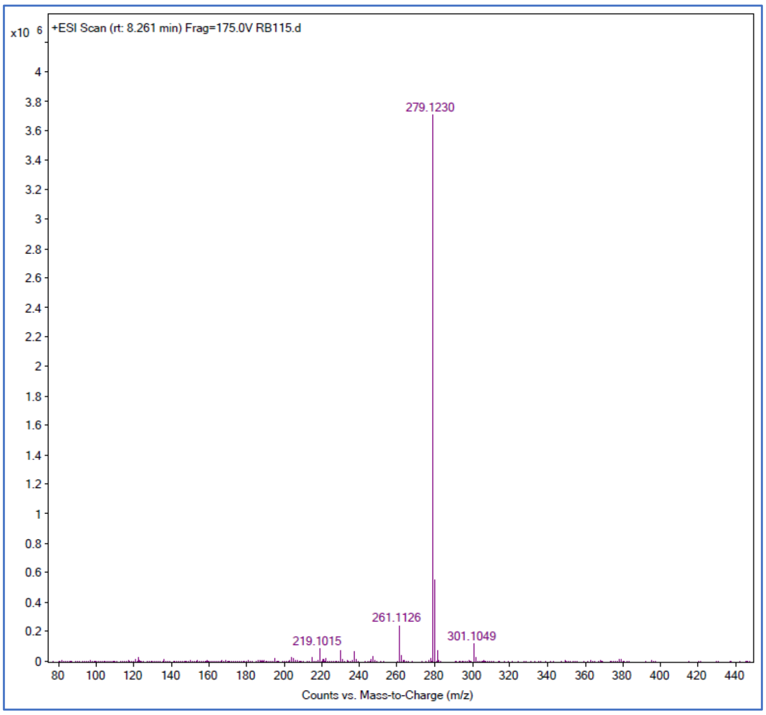

Figure S4. HRMS spectrum of TMBP

DMBP: yield: 21%. ^{1}H NMR (300 MHz, 25 °C, $(CD_3)_2SO$): δ (ppm) = 7.58 (1H, s, H-3), 6.78 (2H, s, H-5), 3.74 (6H, s, H-8), 2.36 and 2.30 (6H, 2s, H-10). ^{13}C NMR (75 MHz, 25 °C, $(CD_3)_2CO$): δ (ppm) = 206.6 and 197.3 (C-1), 147.9 (C-6), 140.6 (C-3), 139.8 (C-7), 138.7 (C-2), 122.8 (C-4), 107.9 (C-5), 55.9 (C-8), 31.5 and 26.1 (C-10). Melting point: 120-123 ± 0.1 °C. TOF MS ES+: $[M+H]^+$ for $C_{14}H_{17}O_5$: *m/z* 265.1076; found: *m/z* 265.1073.

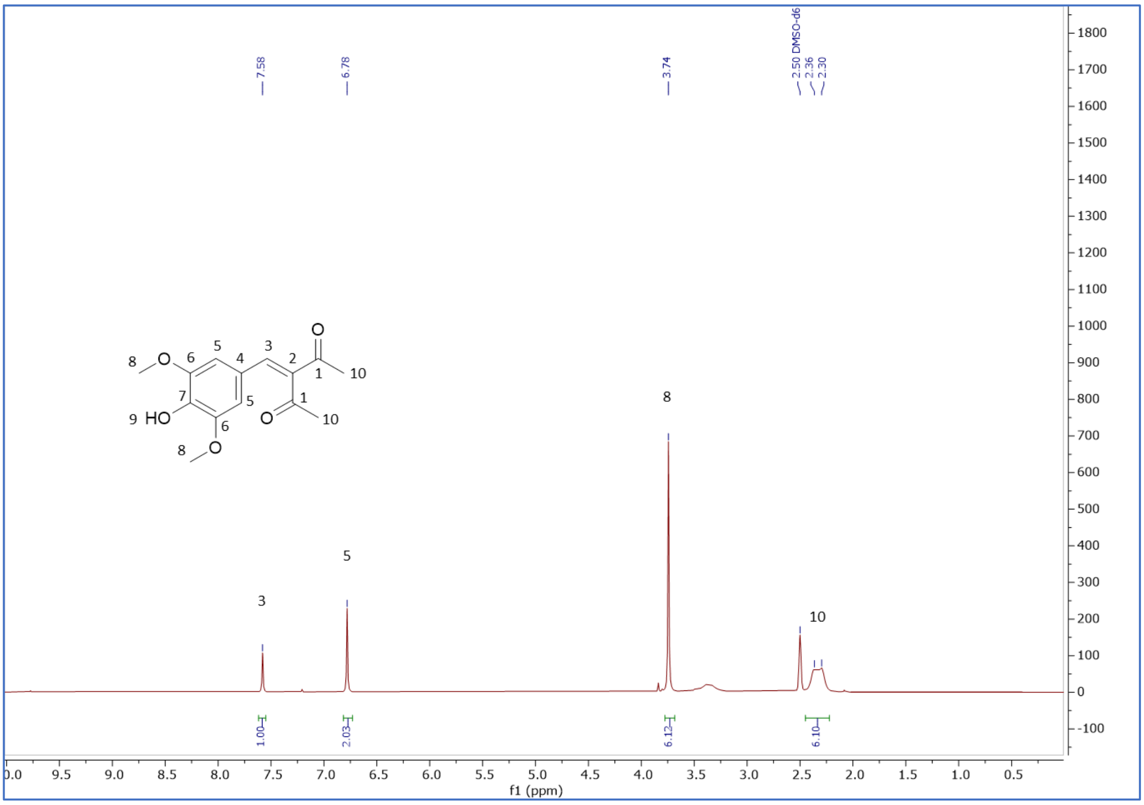

Figure S5. ^{1}H NMR spectrum of DMBP

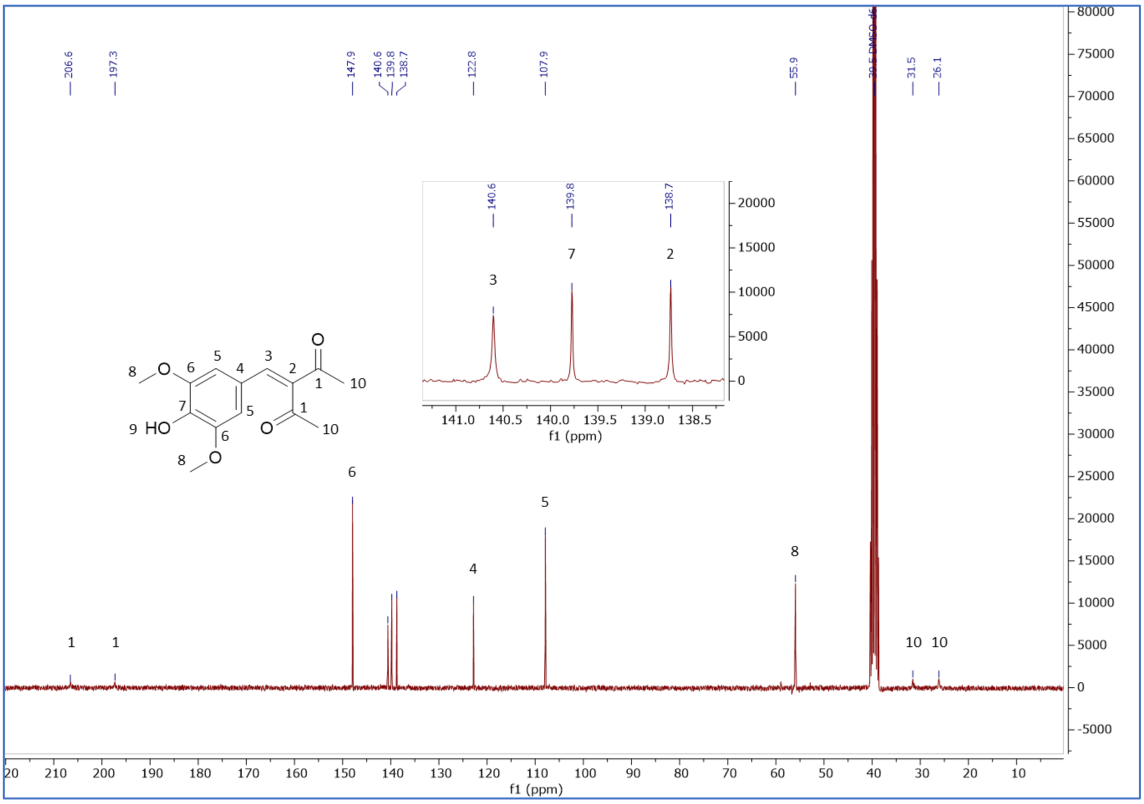

Figure S6. ^{13}C NMR spectrum of DMBP

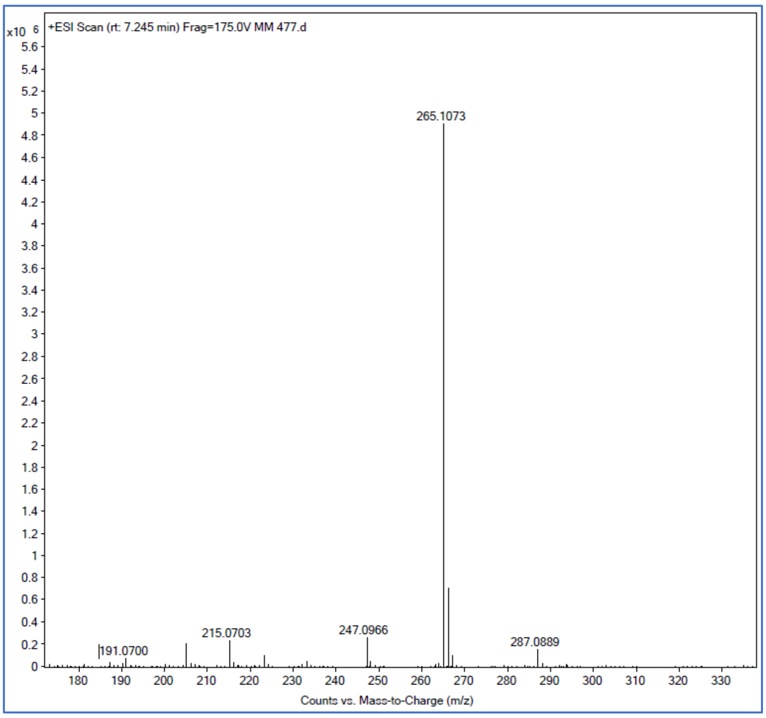

Figure S7. HRMS spectrum of DMBP

C) Computational results

The optimized S_0 and S_1 geometry for both TMBP and DMBP are shown in Figure S8. Following the S_0 geometry optimization, TD-DFT calculations were performed at the optimized ground state geometries to obtain their vertical excitation energies as well as the absorption wavelength presented in Table S1. The orbitals for the three lowest excitation energies are shown in Figure S9 for both TMBP and DMBP. Following this, the geometries of the molecules were optimized in their respective excited state to gain insight into any geometry change that facilitate their relaxation mechanism. The characterization of the excited state of TMBP and DMBP are shown in Figure S10.

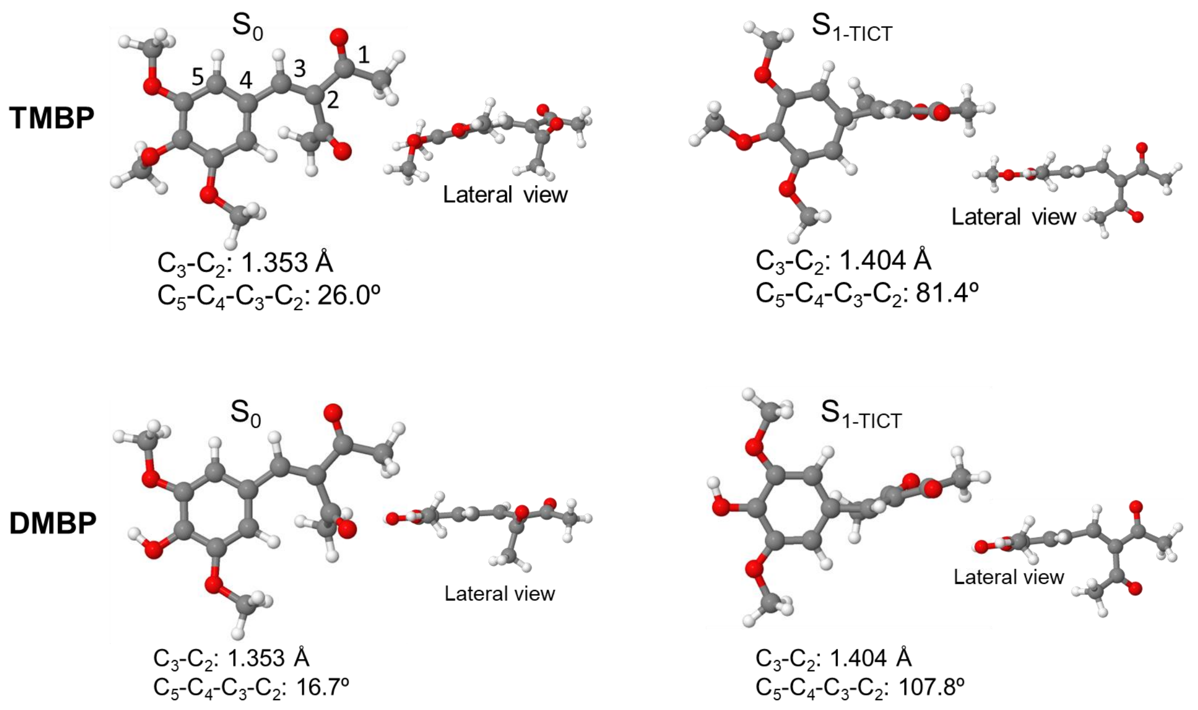

Figure S8. Optimized geometries of TMBP and DMBP in the S_0 and S_1 state calculated at DFT and TD-DFT PBE0/6-311++G** using implicit ethanol. The S_1 geometry showed increased bond length and a twisted geometry at the C_3-C_2 allylic double bond.

Table S1. Vertical excitation energies calculated for the lowest singlet excited state of the optimized S_0 geometries of TMBP and DMBP in implicit ethanol using PBE0/6-311++G**.

Molecule	State	ΔE_{vert} / eV	ΔE_{vert} / nm	Oscillator Strength	State Character
**TMBP**	S_1	3.6097	344	0.0140	ππ*
	S_2	3.6715	338	0.3993	ππ*
	S_3	3.7486	331	0.0136	nπ*
**DMBP**	S_1	3.5203	352	0.4884	ππ*
	S_2	3.7254	333	0.0124	ππ*
	S_3	3.7804	328	0.0000	nπ*

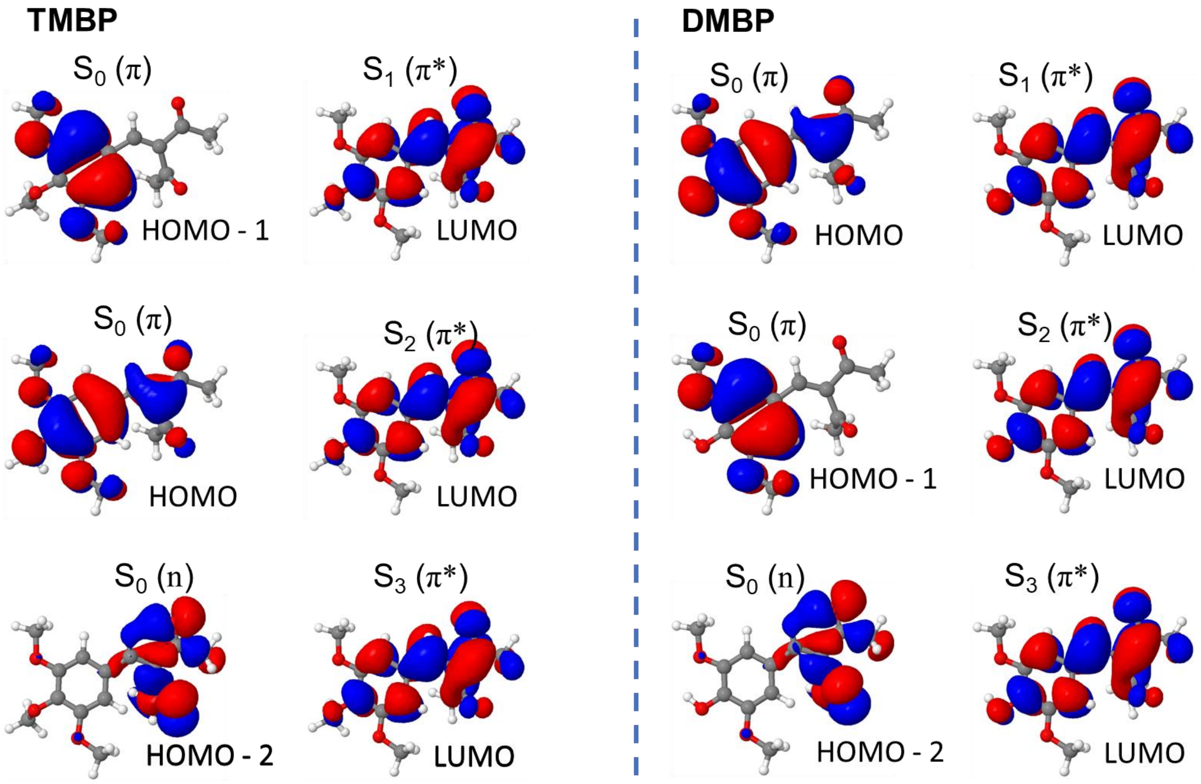

Figure S9. Molecular orbitals (MOs) for the three lowest excitations of TMBP (left) and DMBP (right) calculated at PBE0/6-311++G** using implicit ethanol. For both molecules the excitation at their respective UV absorption λ_{max} corresponds to a HOMO – LUMO transition, i.e. S_2 and S_1 in TMBP and DMBP respectively.

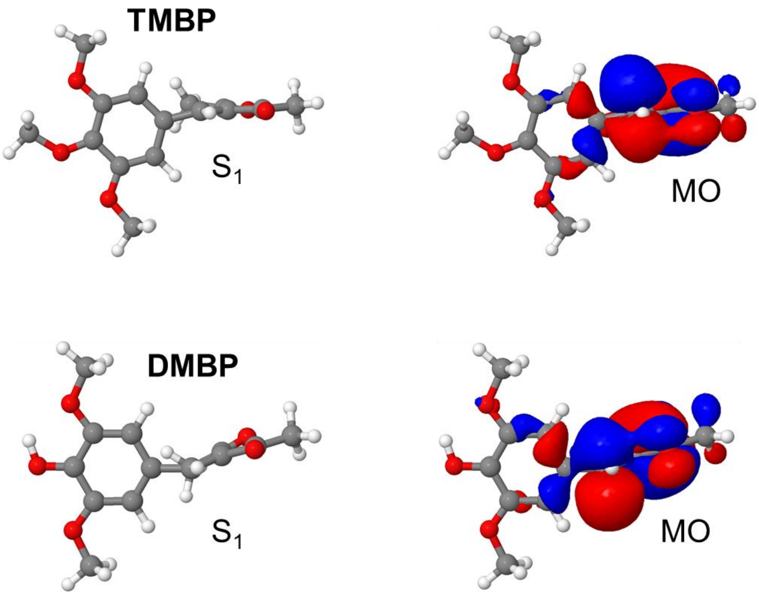

Figure S10. Characterization of S_1 state of TMBP (top) and DMBP (bottom) calculated at PBE0/6-311++G** using implicit ethanol. The MOs (for TMBP and DMBP) revealed a charge transfer character for the optimized structures at the TICT state.

The calculated wavenumbers and the associated vibrational modes for the optimised S_0 geometry of TMBP and DMBP are shown in Table S2. These results guided the assignment of vibrational modes to the experimentally observed FTIR bands. A scaling factor was calculated for each predicted spectrum by using the most intense experimental peak as a reference. This scaling was then applied to the calculated S_0 wavenumbers so that the calculated wavenumber accurately matched the reference experimental peak. This method has previously been employed for similar systems.[1,2]

Table S2. Computed S_0 vibrational wavenumbers and transition intensities for TMBP and DMBP in implicit ethanol using PBE0/6-311++G**. Description of the associated vibrational modes are also described. Atom labels can be found in Figure S8.

Molecule	S_0 wavenumber / cm^{-1}	IR Intensities	Vibrational mode
TMBP	1582	79	C_2=C_3 stretch
	1606	43	C-H bend (ar) + C_2=C_3 stretch
DMBP	1590	72	C_2=C_3 stretch
	1604	34	C-H bend (ar) + C_2=C_3 stretch

D) Kinetic fits

i) TEAS

In this work, a global fitting procedure is employed to determine a set of optimized parameters for the TEA spectra using the Glotaran software package.[3] The various experimental measurables were accounted for by the several components of the algorithm utilized. The TEA spectra obtained from our measurements and presented in the current work are inherently chirped, *i.e.* the position of Δt = 0 varies with each probe wavelength (λ_{pr}) as a result of group velocity dispersion (GVD) artefacts, which is accounted for by including a third order of polynomial in the fitting algorithm. Assuming a sequential ($A \xrightarrow{\tau_1} B \xrightarrow{\tau_2} C \xrightarrow{\tau_3} D\ldots$) kinetic model, the global fitting algorithm in Glotaran models the data for each λ_{pr} and Δt with a superposition Ψ of n components l with the function below:

$$\Psi\ (\lambda, t) = \sum_{l=1}^{n} c_l^{EADS}\ (t, \theta) EADS_l(\lambda) \qquad (1)$$

$c_l^{EAS}\ (t, \theta)$ is a linear combination of exponential decay component l convoluted with the Gaussian instrument response function, and $EADS_l(\lambda)$ is the evolution associated difference spectrum associated with component l. For each set of initial parameters θ, the fit is iterated until it converges. The residuals between the raw data and the fit, determined by the global fitting procedure (and given in Figure S14) demonstrate the quality of the fit.

ii) **TVAS**

For the ground state bleach recovery of the features associated with the TVAS data, an exponential decay function is used to fit the recovery kinetics without the convolution of the IRF using the function:

$$F(\lambda, \Delta t) = I_0 + \sum_{i}^{n} A_i(\lambda)\, e^{\frac{-(\Delta t - t_0)}{\tau_i}} \tag{2}$$

A(λ) is the amplitude of the ith exponential decay component with lifetime τ_i, $\Delta t - t_0$ is the pump-probe time delay, t_0 is the offset recovery time relative to $\Delta t = 0$, I_0 is the baseline signal offset, and n is the number of exponential decay functions required to model the data accurately.

E) TEAS additional data.

The TEAS data presented as a false colour heatmap in the main manuscript is presented as a line plots of mΔOD *vs* probe wavelength at selected pump-probe delay times in Figure S11.

The TEAS data measurement for both TMBP and DMBP in ethanol at a higher concentration of 30 mM as a comparison to the 1 mM measurement are shown in Figure S12. Also shown in Figure S13 are the TEAS data obtained following deposition of the CCT bulk solution of both TMBP and DMBP on the surface of a synthetic skin mimic. These data are at best the same as those obtained in the bulk solution, indicating minimum environment effects on the observed dynamics.

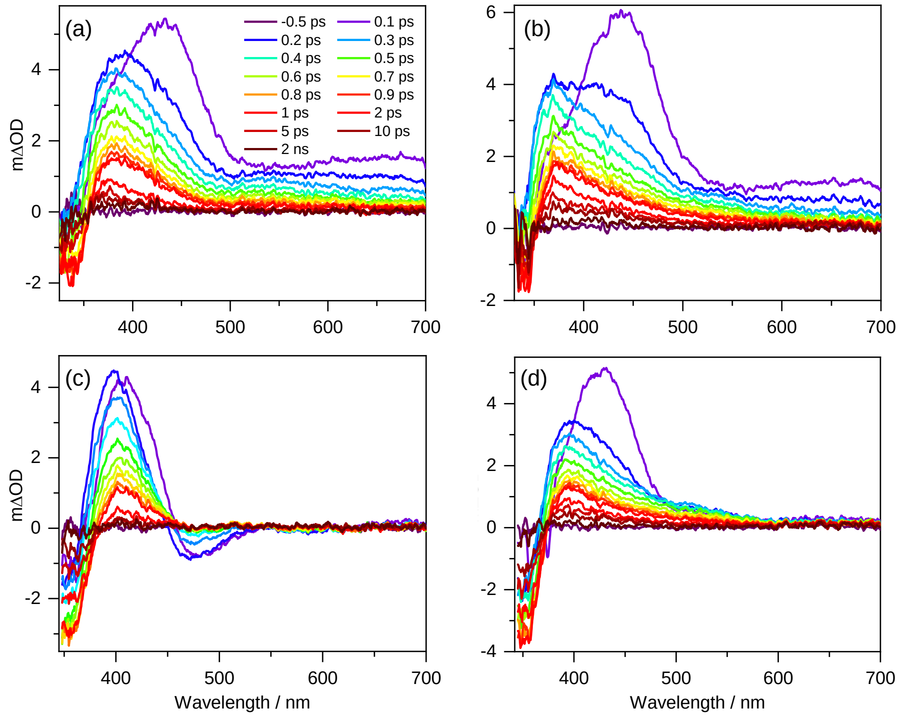

Figure S11. TEA spectra presented as line plots of mΔOD *vs* probe wavelength at selected pump-probe delay times for TMBP in (a) ethanol and (b) CCT; and for DMBP in (c) ethanol and (d) CCT. A colour key for the time delays corresponding to different spectra is shown in panel c.

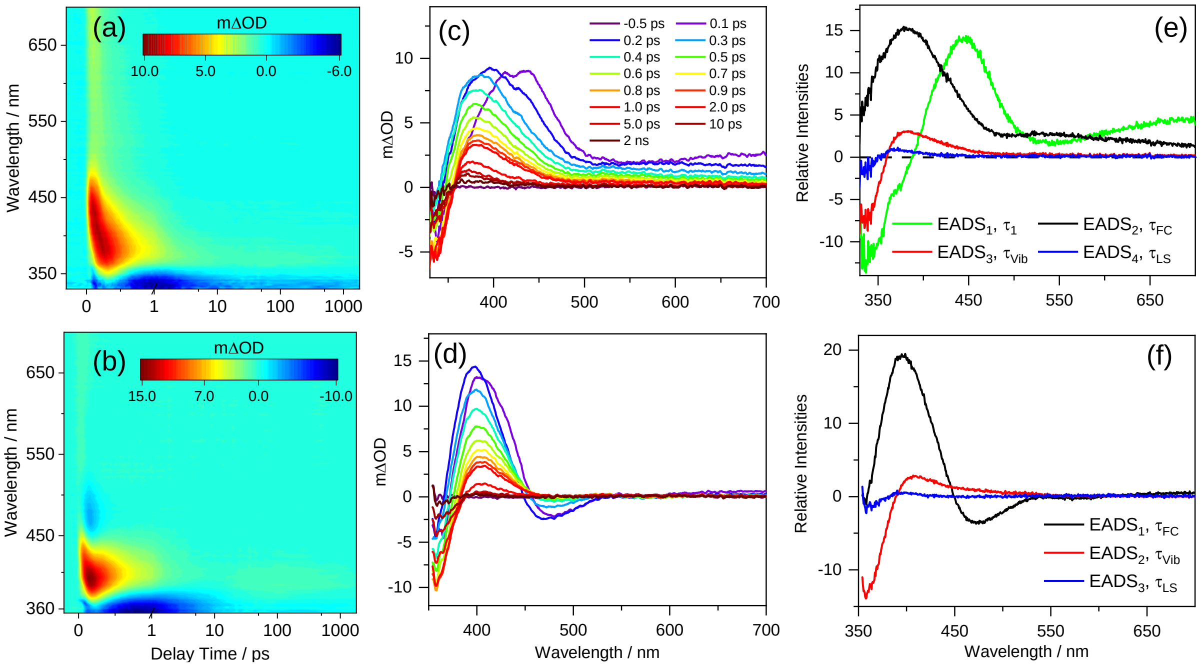

Figure S12. TEA spectra obtained for 30 mM ethanol solution for (a) TMBP and (b) DMBP, both photoexcited at their respective λ_{max}. The TEA spectra are presented as false color maps in both panels. The same data is presented as line plots of mΔOD *vs* probe wavelength at selected pump−probe delay times in panels (c) and (d) for TMBP and DMBP respectively. The EADS produced by the fitting procedure are shown in panels (e) for TMBP and (f) for DMBP.

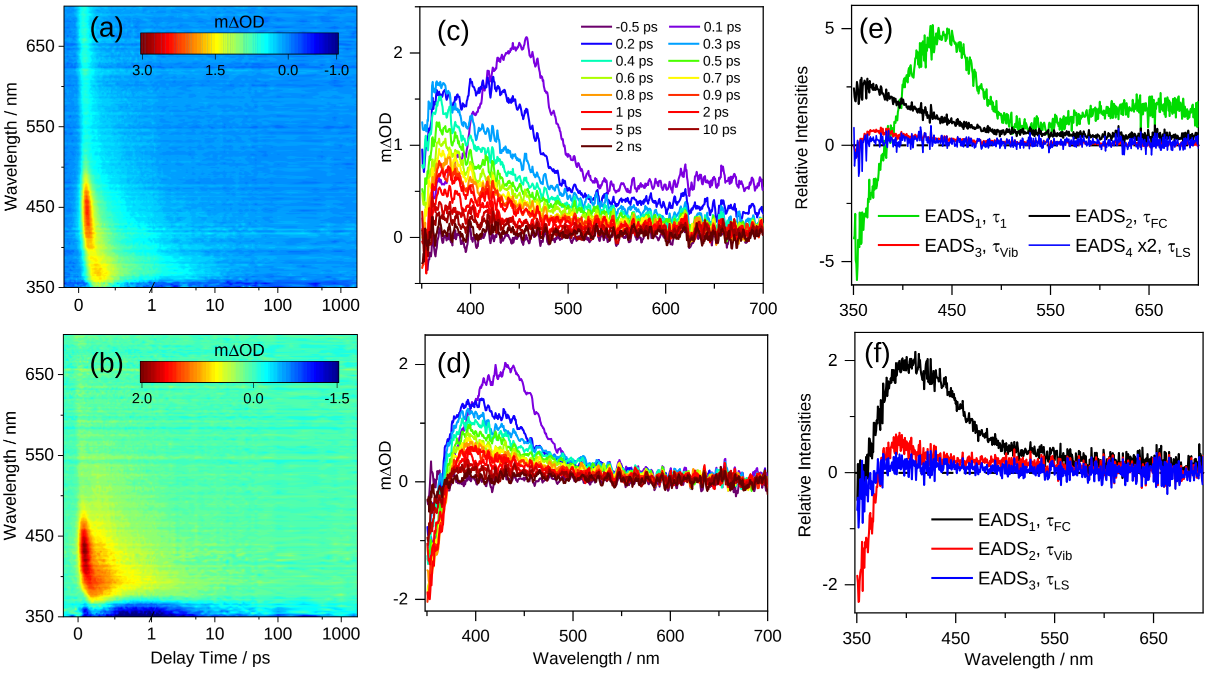

Figure S13. TEA spectra obtained for 30 mM of (a) TMBP VC/CCT and (b) DMBP VC/CCT, both photoexcited at their respective λ_{max} in CCT. The TEA spectra are presented as false color maps in both panels. The same data is presented as line plots of mΔOD vs probe wavelength at selected pump−probe delay times in panels (c) and (d) for TMBP and DMBP respectively. The EADS produced by the fitting procedure are shown in panels (e) for TMBP and (f) for DMBP.

F) Residuals for the fits of TMBP and DMBP in their different solvent environments

The residuals from the sequential global fitting with respect to the raw TEA spectra data (*i.e.,* the difference between the fit and the raw data at each data point) are shown in Figure S14. The small-signal intensities of the residual compared to the raw TEA spectra demonstrate the quality of the fits.

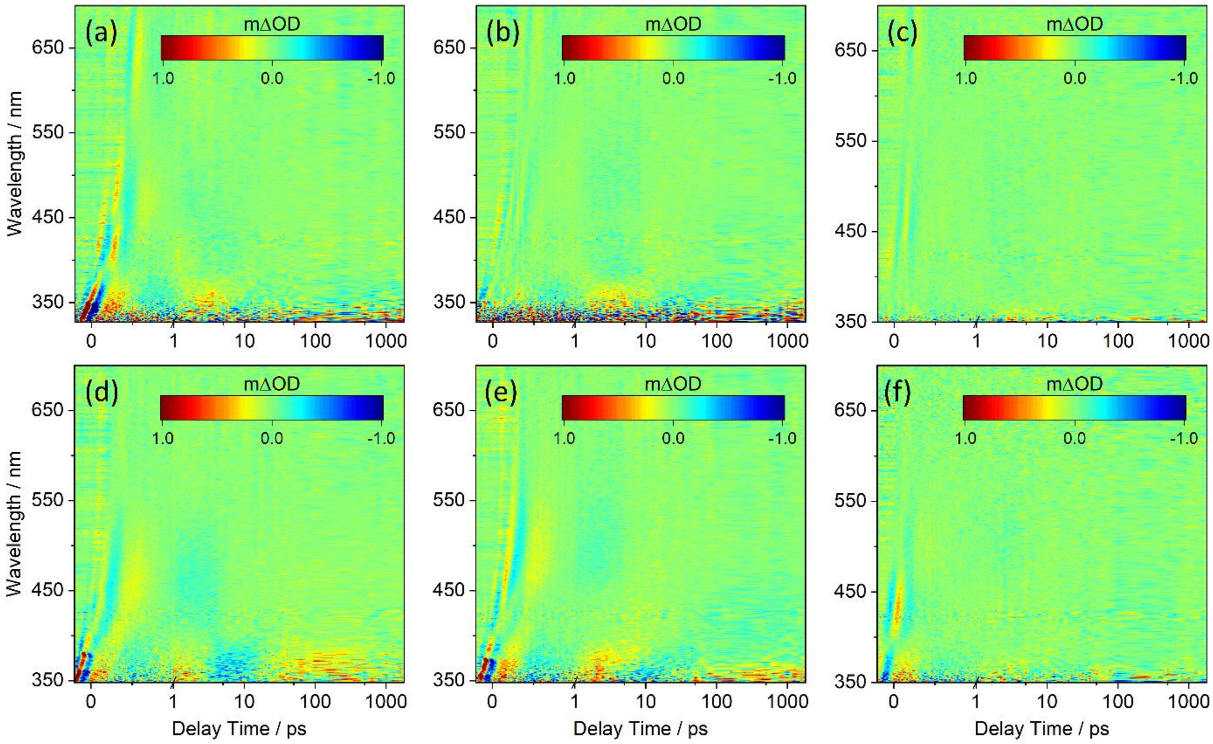

Figure S14. False color heatmap for the residual fit for TMBP in (a) ethanol, (b) CCT and (c) VC/CCT; and for DMBP in (d) ethanol, (e) CCT and (f) VC/CCT.

G) Solvent alone instrument response

The TEAS measurements of the time zero solvent-only scan were recorded to obtain the instrument response function (IRF), which determines the limiting temporal resolution of the present experiments. The data are reported in Figure S15. CCT shows a very weak time-zero response following excitation at 348 nm at the pump-pulse power employed for the experiment. Furthermore, the instrument response function in CCT photoexcited at 348 nm (Figure S15 (d)) has a strong contribution of cross-phase modulation between pump and probe pulses. However, this should not affect the conclusions of the manuscript given the timescales we are investigating in the solute, especially when DMBP is photoexcited in CCT at 348 nm. For this reason, we chose to follow the approach of Kovalenko *et al.*[4], in which we use a frequency-dependent cross-correlation function to

model the IRF in Figure S15. The value obtained for the temporal resolution of the solvent-only time zero response is shown in each panel in Figure S15, and ranges from 70 to 100 fs.

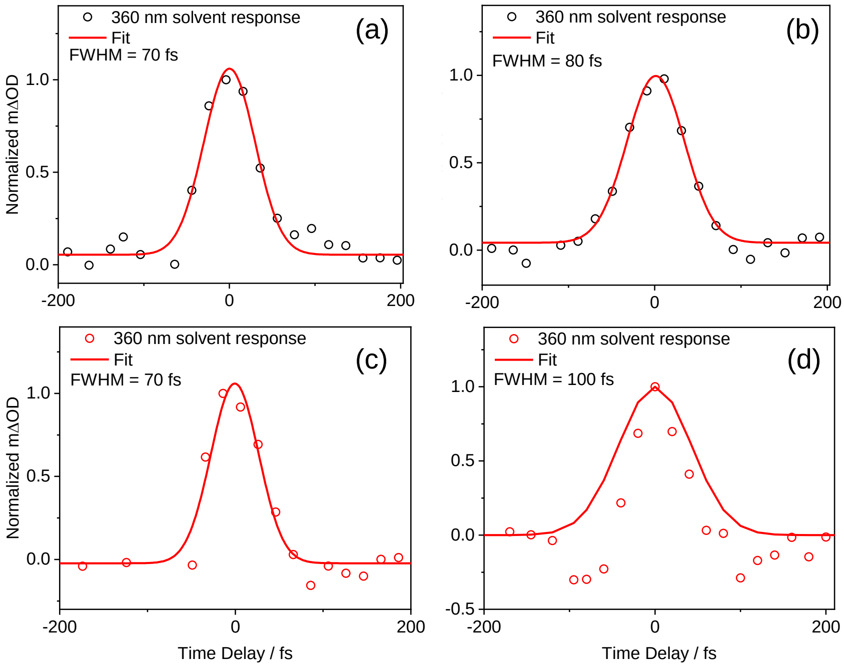

Figure S15. Selected transients for solvent-only time-zero response at a probe wavelength of 360 nm for (a) ethanol and (b) CCT both photoexcited at 321 nm; and (c) ethanol and (d) CCT both photoexcited at 348 nm. The excitation wavelengths for the solvents presented herein correspond to the wavelength at which TMBP (a,b) and DMBP (c,d) samples were excited in each solvent. The extracted full-width half maxima are shown in each panel. These values are used as the instrument response in the corresponding global fit analysis of TEA spectra.

H) 2 ns Transients for TMBP and DMBP in their different solvent environment

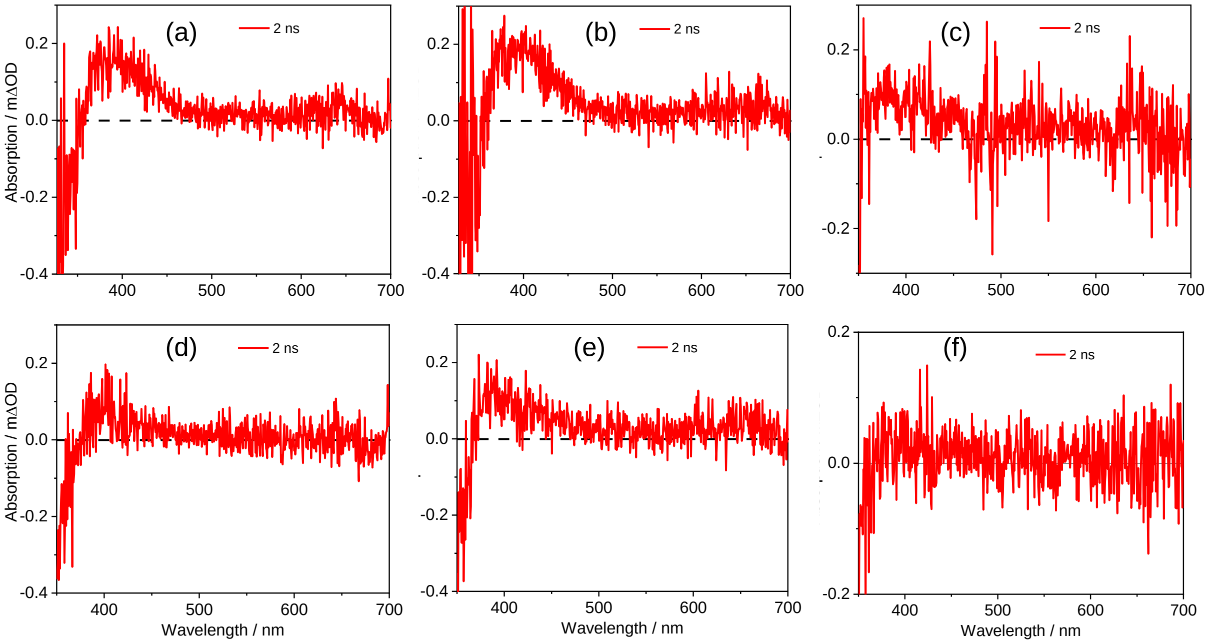

Figure S16. 2 ns transient for TMBP in (a) ethanol, (b) CCT and (c) VC/CCT; and for DMBP in (d) ethanol, (e) CCT and (f) VC/CCT.

I)   Fluorescence lifetime and emission spectra measurements

Figure S17 shows the emission lifetime measurements for both TMBP and DMBP. The absorption and emission spectra for TMBP and DMBP are further reported in Figure S18. To add, we have also carried out emission lifetime measurements after flushing nitrogen through the samples; the data return similar lifetimes to those reported in Figure S17. Taken together (the short emission lifetimes and small Stokes shifts), we propose that the emission we observe is from the singlet state and that the long-lived component in our TEA spectra is trapped population in the S_1 state. To note, we cannot rule out triplet state emission due to limitations to our experimental setup.

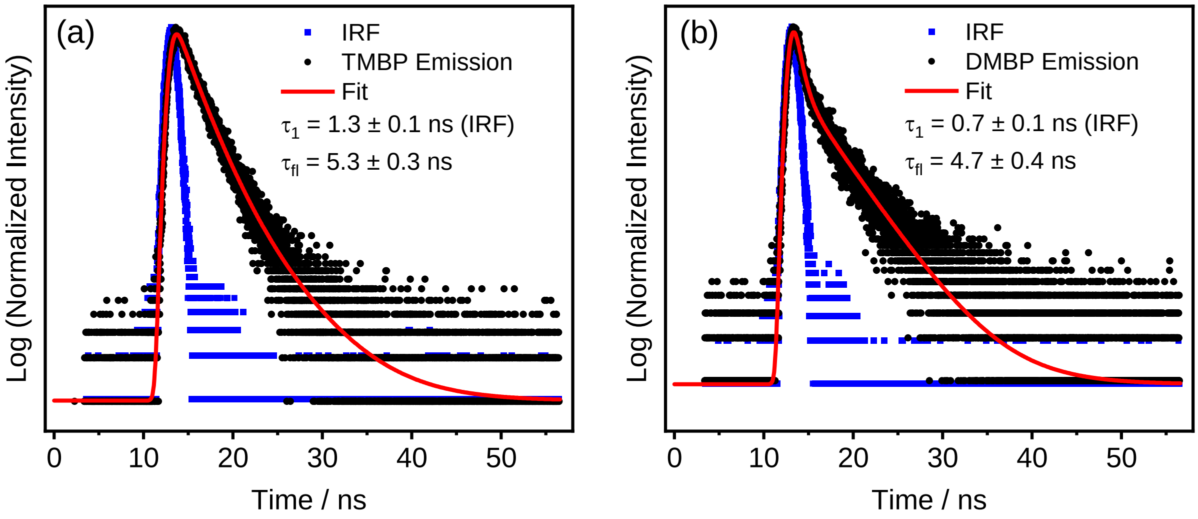

Figure S17. Fluorescence lifetime measurements for (a) TMBP, and (b) DMBP. In both cases, data are shown as black circles, with the red line being a kinetic fit from which fluorescence lifetime is obtained. The instrument response for these measurements is also shown as blue squares.

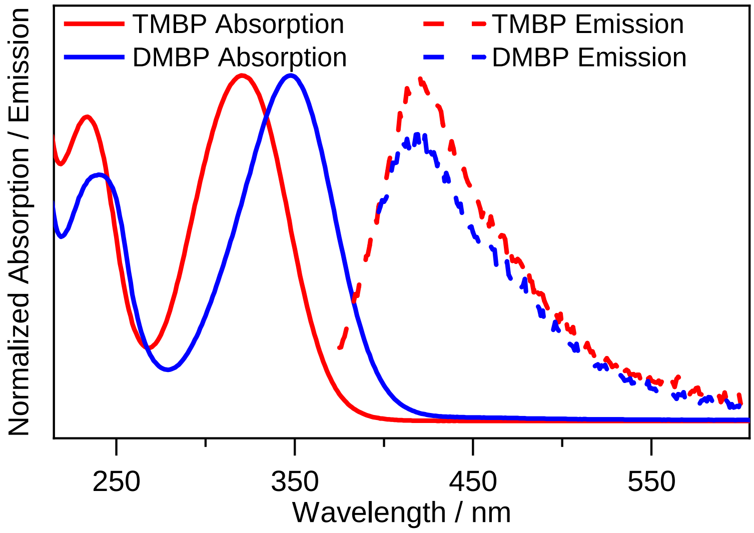

Figure S18. Normalized absorption spectrum of TMBP (solid red line) and DMBP (solid blue line), and the normalized emission spectrum of TMBP (dash red line) and DMBP (dash blue line) in

ethanol. Each absorption spectrum is normalized to the TMBP and DMBP λ_{max} absorbance; for the emission spectra, each spectrum is normalized to the peak of TMBP emission.

J) FTIR spectra

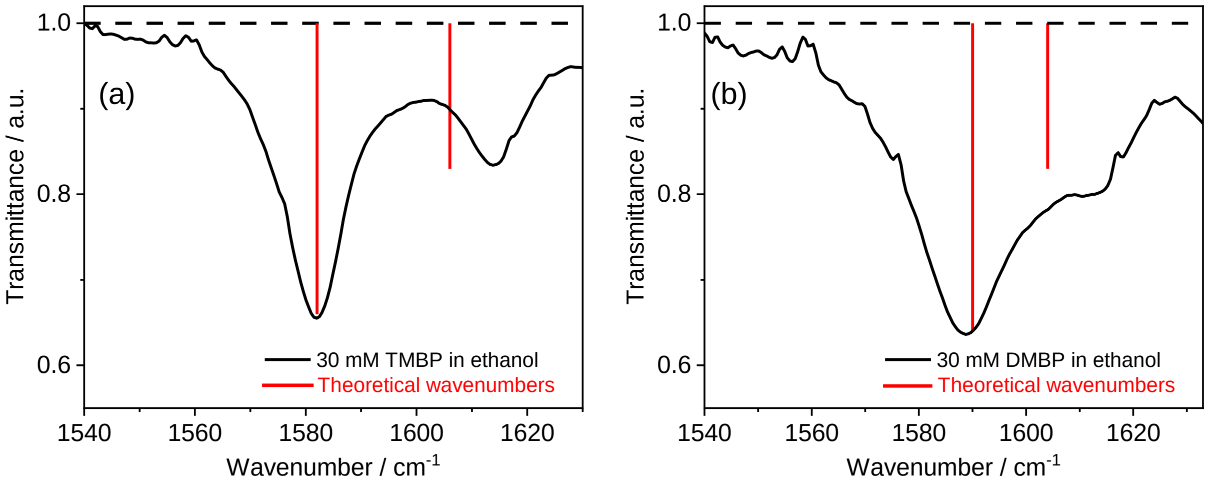

Figure S19. Experimental steady-state FTIR spectra of 30 mM solution of (a) TMBP and (b) DMBP, both displayed as a solid black line. Overlaying the FTIR spectra are wavenumbers (red vertical lines) of the vibrational modes (see below) predicted at the PBE0/6-311++G** level of theory with an implicit ethanol solvent model that are scaled with a scaling factor of 0.9814 for TMBP and 0.9646 for DMBP. The amplitudes of the red vertical lines reflect the IR transition intensities returned by the calculation and scaled to the most intense peak in both molecules. Frequency calculations suggest the vibrational features displayed correspond to the allylic C=C stretching at 1582 (1590) cm^{-1} and aromatic C–H bending and C=C stretching at 1606 (1604) cm^{-1} for TMBP (DMBP).

K) Steady-state photostability of TMBP and DMBP in CCT.

Additional photostability data obtained for TMBP and DMBP in CCT are reported in Figure S20. These data revealed that TMBP maintained its high photostability with ~2%; in contrast, a significant reduction (~30%) in the absorbance of DMBP is observed. Possible sources of the reduction in absorption could include the formation of molecular photoproduct that absorbs at a different wavelength. However, the 2 ns transient absorption profile does not show any significant difference when compared to that of TMBP (Figure S16). Hence, it is unlikely that any photoproducts different from those formed in TMBP dissolved in CCT following irradiation are formed in DMBP. Another plausible explanation could be that upon photoexcitation of CCT, it becomes a photoacid which could then react with DMBP resulting in the decrease in the absorption spectrum (Figure S20b).

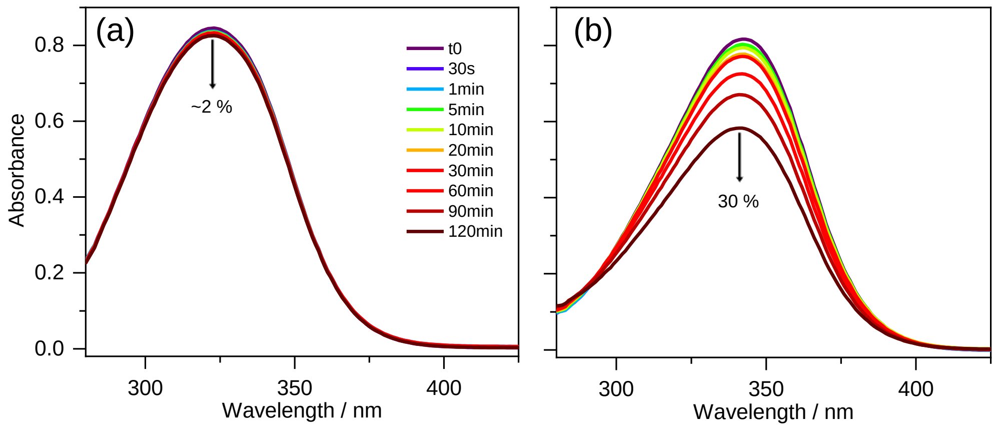

Figure S20. UV-visible spectra before and at various times during 2 hrs. irradiation with solar simulator in a 1 mm cuvette for (a) TMBP and (b) DMBP in CCT. The downward arrows denote the observed decrease in λ_{max} absorbance over 2 hrs. of irradiation.

L) UV/Visible difference spectrum.

Figure S21 shows the difference spectra obtained by subtracting the pre-irradiated absorption spectrum from post-irradiated (with a solar simulator) absorption spectrum for TMBP and DMBP. For both samples in ethanol, there is a decrease in absorption at ~320 and ~340 nm for TMBP and DMBP respectively, in both solvent environments which corresponds to reduction of the absorption peak. As described in the main manuscript, the difference spectra do not closely match the absorption in the 2 ns TAS reported in Figure S16 (for TMBP and DMBP), lending to the conclusion that trapped excited state population in the singlet state is the main source of incomplete GSB recovery. Furthermore, the additional negative peak observed at ~450 nm in the difference spectrum of DMBP in ethanol (Figure S21c) originates from the photodegradation of a phenolate form of DMBP which is present in the UV visible spectrum of DMBP in ethanol before irradiation.

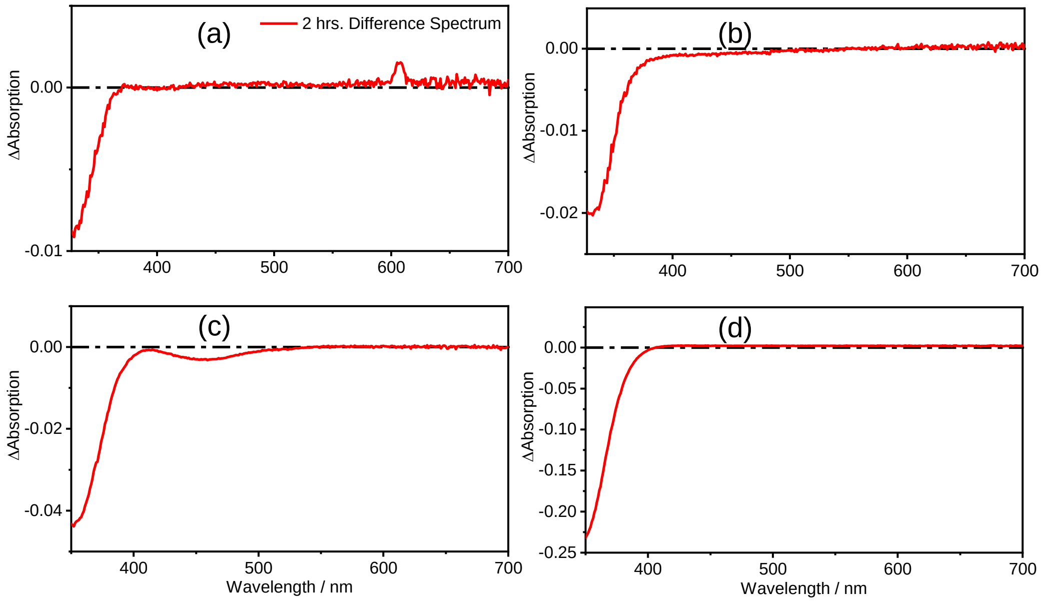

Figure S21. Difference spectra between 2 hr solar like irradiation and before irradiation for TMBP in (a) ethanol, (b) CCT; and for DMBP in (c) ethanol, and (d) CCT.

M) ^{1}H NMR of TMBP and DMBP before and after solar irradiation

In an attempt to investigate the possible formation of any long-lived photoproducts upon UV excitation of both TMBP and DMBP, ^{1}H NMR spectra of separate samples prepared to 0.5 M were recorded in deuterated ethanol (ethanol-d_6) before and after 5 hours of continuous irradiation under a solar simulator with irradiance equivalence to 7 suns (7000 W/m^2). Irradiation was achieved in a 1 cm cuvette. As shown in Figure S22, the data did not reveal any obseveable new peaks after sample irradiation, suggesting little or no photoproduct formation. However, we note that the integration of the peak assigned to hydroxy proton (-OH, labelled 9) in DMBP varies between before (1.46) and after (4.63) irradiation. Integration of all other peaks however remained the same before and after irradiation. This could result from a number of reasons, including (*i*) effect of water molecules in the solvent due to the hygroscopic nature of the solvent, (*ii*) effect of hydrogen bonding between ethanol and the phenolic group, or (*iii*) formation of potential photoproduct.

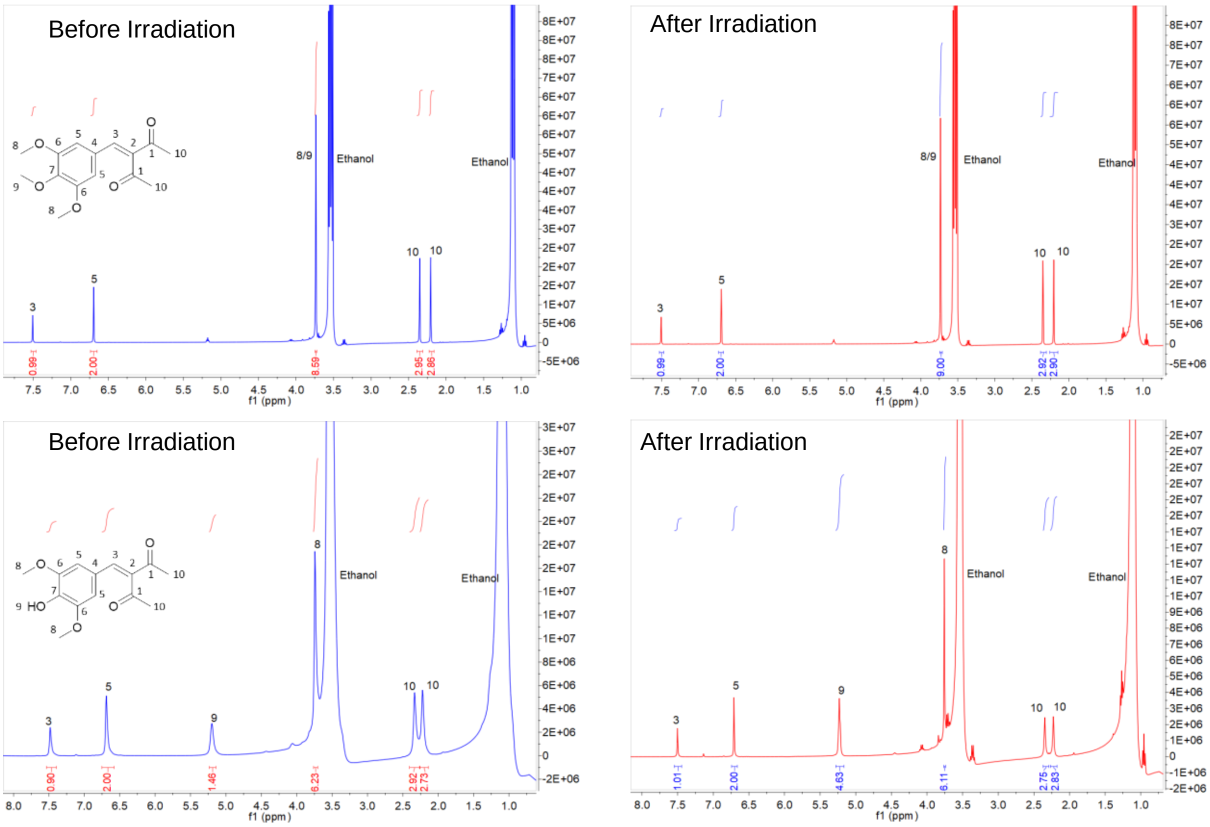

Figure S22. ^{1}H NMR spectra of TMBP (top panel) and DMBP (bottom panel); before (blue line) and after 2 hr of solar-like irradiation (red line).

N) Supplementary References

1. Turner, M. A. P.; Turner, R. J.; Horbury, M. D.; Hine, N. D. M.; Stavros, V. G., Examining solvent effects on the ultrafast dynamics of catechol. *J. Chem. Phys.* **2019,** *151* (8), 084305.
2. Whittock, A. L.; Turner, M. A. P.; Coxon, D. J. L.; Woolley, J. M.; Horbury, M. D.; Stavros, V. G., Reinvestigating the Photoprotection Properties of a Mycosporine Amino Acid Motif. *Front. Chem.* **2020,** *8* (574038), 574038.
3. Snellenburg, J.; Laptenok, S.; Seger, R.; Mullen, K. M.; Van Stokkum, I. H. M., Glotaran: A Java-based graphical user interface for the R package TIMP. *J. Stat. Softw.* **2012,** *49*, 1-22.
4. Kovalenko, S. A.; Dobryakov, A. L.; Ruthmann, J.; Ernsting, N. P., Femtosecond spectroscopy of condensed phases with chirped supercontinuum probing. *Phys. Rev. A.* **1999,** *59* (3), 2369.
